# Per‐Channel Complex $B_{1}^{+}$ Mapping for Parallel Transmit Ultra‐High Field MRI

**DOI:** 10.1002/nbm.70425

**Published:** 2026-11-03

**Authors:** Ye Yuan, Jianxiang Chen, Christopher T. Rodgers

**Affiliations:** ^1^ Wolfson Brain Imaging Centre University of Cambridge Cambridge UK

**Keywords:** $B_{1}^{+}$ mapping, parallel transmission, pTx, pulse design, ultra‐high field MRI

## Abstract

Ultra‐high field (UHF, B_0_ ≥ 7 T) MRI provides substantial gains in signal‐to‐noise ratio, spatial resolution and contrast. Routine clinical deployment has been limited by challenges of transmit field ($B_{1}^{+}$) inhomogeneity and specific absorption rate (SAR). Parallel transmission (pTx) can mitigate these challenges but it requires per‐channel complex $B_{1}^{+}$ maps to design effective pTx pulses. We review established and emerging approaches to obtain these maps. We begin by summarising the underlying physical principles and theory. Then we describe methods for magnitude $B_{1}^{+}$ mapping such as actual flip‐angle imaging (AFI), saturation‐prepared $B_{1}^{+}$ mapping (e.g., satTFL) and dual refocusing echo acquisition mode (DREAM) that are the basis of widely‐applied per‐channel complex $B_{1}^{+}$ mapping methods, as well as methods of pedagogical and/or historical relevance. Next, we describe how *relative* $B_{1}^{+}$ mapping with low‐flip angle gradient‐recalled echo (GRE) scans can determine the relative *phases* (and relative magnitudes) of $B_{1}^{+}$ for each channel. We recap the important *interferometric* approach that is now widely used. We explain how magnitude and phase maps are combined into complex per‐channel $B_{1}^{+}$ maps. We discuss remaining arbitrary factors such as the position‐dependent transmit reference phase. We discuss recent practical implementations such as Sandwich TFL, 3DREAM, compressed‐sensing SA2RAGE and respiratory‐resolved $B_{1}^{+}$ mapping. We explore the workflow improvements that could accrue if per‐channel $B_{1}^{+}$ maps do not need to be acquired for every subject. This includes deep‐learning–based approaches to *synthesize* $B_{1}^{+}$ maps from the normal localiser scans, the universal pulses (UPs) approach to precalibrate pulses and variants such as standardised universal pulses (SUPs), Fast‐Online Customised (FOCUS) and machine‐learning online customisation. We hope to encourage greater openness and harmonisation of these critical calibration steps for parallel transmit pulse design. This will become increasingly important as 7‐T pTx MRI is adopted for multisite cross‐vendor clinical research studies and to deliver the full scientific potential of new extremely high field human MRIs.

## Graphical abstract

Transmit‐field inhomogeneities beset ultra‐high field (UHF) MRI scans using single‐channel transmission or circularly polarised (CP) mode. Parallel transmission mitigates these artefacts but requires accurate subject‐specific per‐channel complex (magnitude and phase) $B_{1}^{+}$ maps. We explain the theory of per‐channel complex $B_{1}^{+}$ mapping and review published implementations. We compare accuracy, dynamic range and robustness. We highlight innovations such as universal pulses, deep‐learning synthesis and accelerated 3D methods. We hope this review will help demystify this important aspect of UHF MRI workflows.

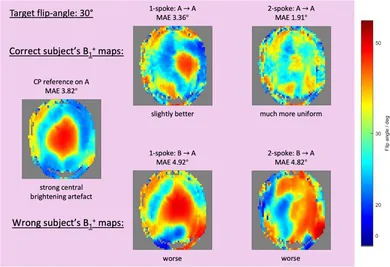

## 1 Introduction

Ultra‐high field (UHF, B_0_ ≥ 7 T) MRI scanners are now installed at more than 100 sites worldwide. These systems offer improved spatial resolution and improved signal‐to‐noise ratio (SNR) compared with 1.5‐ and 3‐T scanners. They also offer more helpful image contrast for some important sequences, such as blood oxygenation level dependent (BOLD) for functional imaging studies [1] or effective transverse relaxation rate R_2_* mapping.

Human UHF MRI began in 1998 with an 8‐T MRI at Ohio State University [2], followed shortly after by a custom‐built 7 T at the University of Minnesota in 1999 and the first commercial 7‐T MRI at Massachusetts General Hospital (MGH) in 2002 [3]. By 2015, more than 50 research sites had 7‐T MRIs, which proved their value for neuroimaging—especially for sub‐millimetre structural and functional imaging studies [4, 5]. As technical challenges were overcome, 7‐T MRI was shown to be superior to 3 T in clinical research studies, such as in epilepsy [6] and multiple sclerosis [7, 8]. Single transmit 7‐T MRIs with ce/FDA approval for clinical diagnostic imaging were released by Siemens (2017) and GE (2020), and since 2024 with *parallel transmission (pTx)*. This reflects a growing momentum towards clinical applications in neurology [9].

Pioneering theoretical and experimental studies demonstrated that while 7‐T MRI increases signal‐to‐noise ratio, this is accompanied by non‐uniform excitation and localised tissue heating [10, 11, 12, 13]. This arises because 7‐T MRI scanners operate at higher frequency (298 MHz) than 1.5‐ or 3‐T MRI scanners. Human tissue has high relative permittivity (ε_r_ ≈ 50–80), so this corresponds to a ~10‐cm wavelength for the radiofrequency (RF) transmit field ($B_{1}^{+}$) in vivo, which is comparable to the size of the head and/or body and hence causes strong interference effects. Different human tissues have various conductivities (*σ* ≈ 0.3–1 S/m) which cause subject‐specific attenuations and propagation delays (i.e., RF phase). Uncontrolled, these produce spatially varying flip angles that cause ‘signal voids’—dark patches in images that could hide pathology or otherwise confuse reading of the scan [14]. The same physics also contributes to localised RF heating, that is, local specific absorption rate (SAR) hotspots.

pTx utilises multiple RF transmit channels to provide additional spatial degrees of freedom to mitigate non‐uniformities in flip angle and SAR [15]. Most pTx pulse design methods require access to accurate complex transmit sensitivity maps for each channel (per scan or during setup). Therefore, rapid and reliable $B_{1}^{+}$ mapping methods are critical to successful pTx implementations.

## 2 Parallel Transmission (pTx)

### 2.1 Hardware Overview

Modern ‘dynamic’ parallel transmit systems use coils with multiple transmit elements, typically eight in commercial 7‐T MRI systems, as illustrated in Figure 1B [18, 19]. For comparison, a conventional single transmit coil is shown in Figure 1A. Each transmit element is driven independently by its own RF waveform generator and RF amplifier. In addition, the forward and reflected RF signals of each channel can be monitored using directional couplers, as illustrated in Figure 1C [20, 21]. This architecture allows independent RF waveforms to be applied to each transmit channel, substantially increasing the degrees of freedom available for excitation design. Consequently, it enables advanced applications such as slice‐by‐slice RF shimming in interleaved multislice acquisitions, multispoke RF pulses and spatially tailored excitations to improve flip‐angle homogeneity and facilitate SAR management. For basic imaging, a circularly polarised (‘CP’ or ‘CP+’) mode is often used, wherein all channels have equal amplitude and ‘clock face’ relative phases (0°–315° for an 8‐element coil) [16, 17].

**FIGURE 1:**
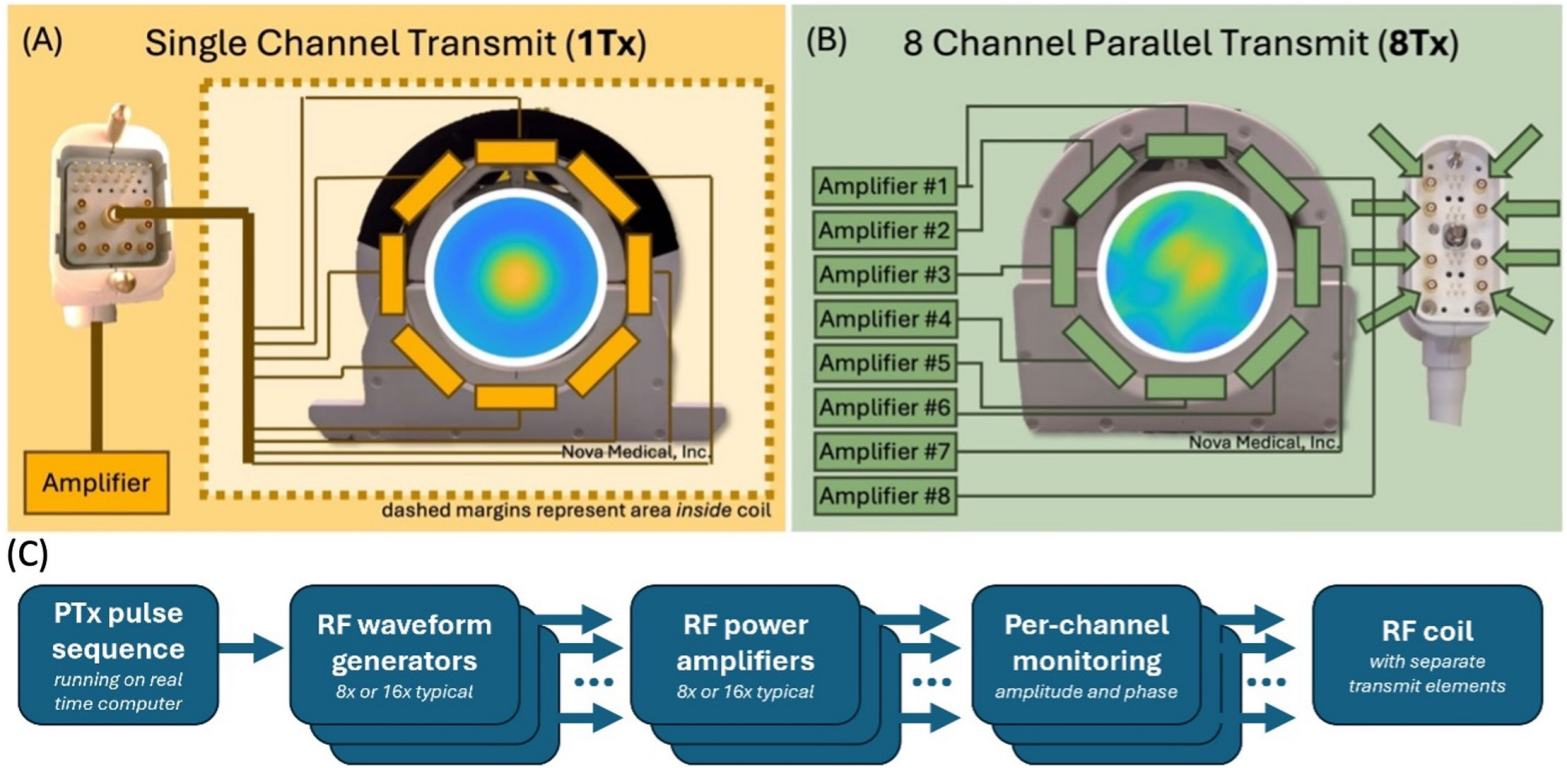
An example of (A) an eight‐rung single‐transmit (1Tx) birdcage coil with all rungs driven by a single RF amplifier. The birdcage end‐rings physically impose equal amplitude current in each rung, with ‘clock face’ relative phases (0° to 315°). This is often referred to as the ‘Circularly Polarised’ (‘CP’ or ‘CP+’) birdcage mode [16, 17]. (B) An eight‐channel pTx coil with eight separate elements (and no end‐rings) driven by independent RF amplifiers, with the i^th^ element having a phase of φ_i_ and amplitude $\alpha_{i}$. This arrangement provides additional degrees of freedom that can be used to mitigate $B_{1}^{+}$ inhomogeneity. If equal amplitudes and ‘clock face’ relative phases are chosen, that is, CP mode, coil performance is often similar to the single‐transmit birdcage (A). (C) Typical system architecture supporting parallel transmission and safety monitoring.

In contrast, some early pTx implementations used a simpler approach, often referred to as ‘static parallel transmission’ or ‘RF shimming’. A single RF waveform generator was connected, via a power splitter, to a multichannel programmable phase and gain modulator [22, 23]. This configuration enabled fixed, channel‐specific amplitudes and phases to be applied throughout an MRI sequence, thereby allowing static RF shimming [24]. However, unlike modern dynamic pTx systems, it could not generate independently varying RF waveforms across the transmit channels and therefore did not support more advanced applications such as slice‐specific RF shimming or multispoke excitation.

### 2.2 Role of pTx Pulse Design Algorithms

Figure 2B,C shows the examples of static and dynamic pTx pulses compared with CP‐mode pulse in Figure 2A. pTx pulse sequences utilise the additional degrees of freedom provided by this hardware. This is used to improve image *fidelity*, preventing signal voids from obscuring parts of the brain and achieving the desired contrast everywhere. It can also improve RF safety management by redistributing RF power deposition and mitigating local SAR hotspots through spatially tailored RF excitation strategies, while satisfying SAR constraints during pulse design. Both homogeneous excitation for high diagnostic image quality and effective SAR management are vital for maximising the benefit‐to‐risk ratio of UHF MRI.

**FIGURE 2:**
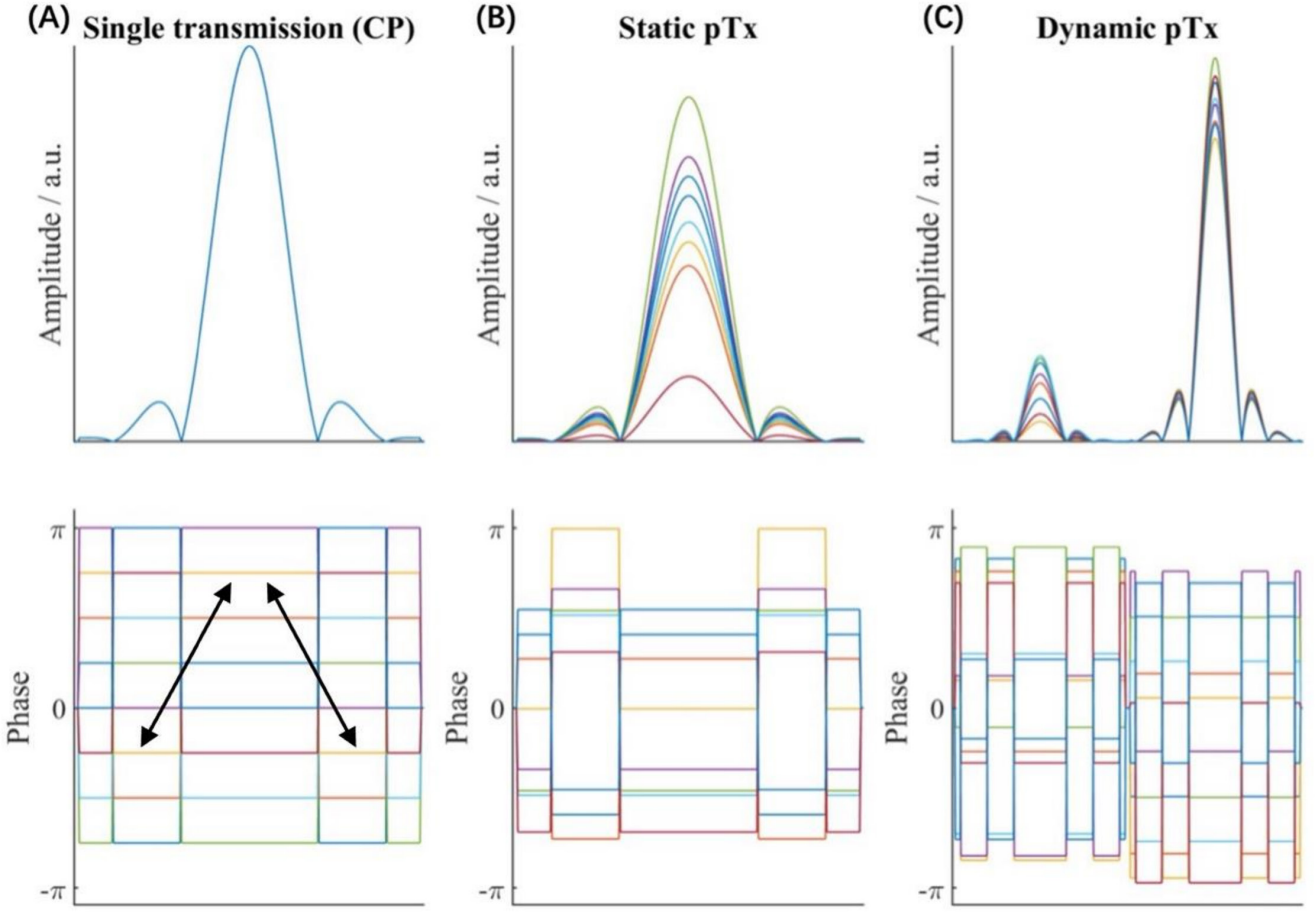
(A) CP‐mode Hann windowed sinc pulse with equal phase differences between adjacent channels. Phase is shown by the various coloured lines. Note the π phase shift for the negative side‐lobes in the sinc envelope. Note that +π and −π phases are equivalent; we choose to plot the purple curve at +π. (B) Static pTx pulse with channel‐specific complex weightings that remain constant (apart from the π phase shift for the negative side‐lobes in the sinc envelope). (C) Dynamic 2‐spoke pTx pulse with different waveforms on each transmit channel. Note ±π phase shift for side lobes relative to main lobe in sinc‐like pulse. Figure reproduced from [25] with permission.

Most pTx design algorithms require subject‐specific per‐channel *complex* $B_{1}^{+}$ maps that show the spatial variation in $B_{1}^{+}$ amplitude and phase for a specified input, for example, 1 V_RMS_ at 0° phase relative to the local oscillator. UHF $B_{1}^{+}$ maps vary from subject‐to‐subject depending on coil loading, dielectric resonances and the propagation of RF through the subject. Traditionally they are measured using a $B_{1}^{+}$‐mapping prescan [26]. Accurate subject‐specific $B_{1}^{+}$ maps are critical for effective RF shimming and homogeneous excitation, as illustrated in Figure 3, where it can be seen that applying the wrong $B_{1}^{+}$ information can impair FA uniformity. Hence, there has been significant attention to improving $B_{1}^{+}$ mapping's speed and accuracy.

**FIGURE 3:**
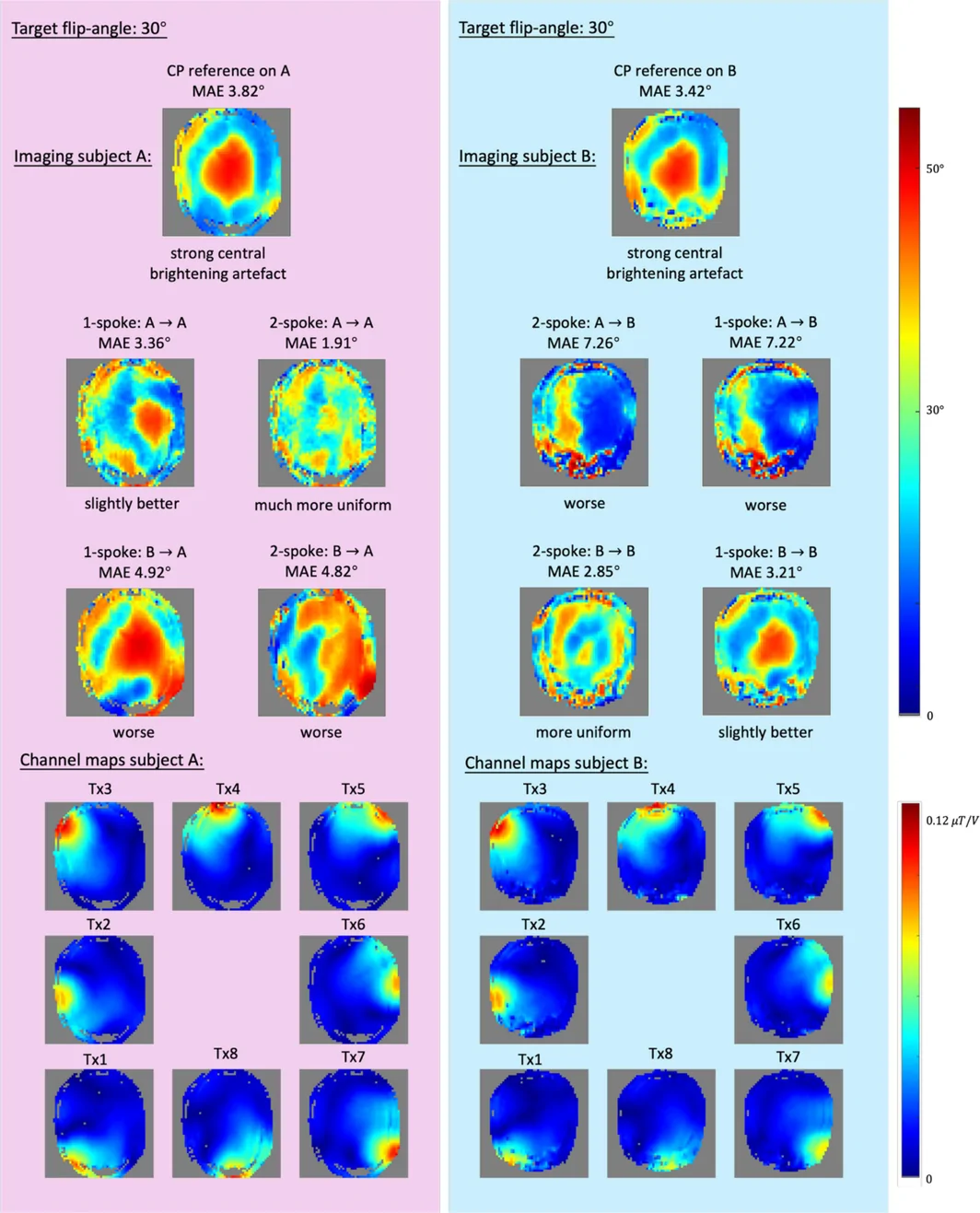
Subject specificity of parallel‐transmit (pTx) RF shimming. Top panels show Bloch‐simulated flip‐angle distributions for two subjects (A and B), comparing circularly polarised (CP) excitation, one‐spoke (i.e., RF shimmed) excitation, and two‐spoke pulse excitation for a uniform 30° target flip angle. Left: results for imaging subject A, including CP excitation; one‐ and two‐spoke pulses designed using subject A's channel maps and applied to A (A → A); and pulses designed using subject B's channel maps but applied to A (B → A). Right: equivalent results for imaging subject B, comparing subject‐matched designs (B → B) with pulses designed from subject A's channel maps (A → B). Each flip‐angle panel reports the mean absolute error (MAE) in degrees within the excitation region. The bottom panels show the individual 8 transmit‐channel (Tx1–8) maps for subjects A and B, illustrating the subject‐dependent spatial sensitivity profiles used for pulse optimisation. The channel maps differ between the two subjects, and consequently, pulses optimised for one subject are not generally appropriate for the other. Subject‐matched designs improve uniformity, whereas applying a pulse optimised from the other subject's channel maps degrades performance compared with CP excitation. These results demonstrate the importance of subject‐specific $B_{1}^{+}$ maps for pTx pulse optimisation.

pTx design algorithms are the topic of active research and have been ably reviewed elsewhere [15, 26]. MRI vendors provide closed‐source toolkits including the pTx Pulse Design (PPD) toolbox (Siemens), EPIC pTx extensions (GE) and GyroTools (Philips). Table S1 in the Supporting Information summarises currently available open‐source pTx pulse design codes. FastPtx, SigPy.RF and PulPy provide integrated toolboxes. Most other codes implement a specific algorithm to accompany a paper. There is a need for a full‐featured, multivendor, open‐source pTx design package, perhaps implemented around the Pulseq standard [27].

### 2.3 Importance of $B_{1}^{+}$ Phase

pTx pulse design requires knowing the complex transmit sensitivity (magnitude and phase) for every channel. This is because nuclear spins experience the coherent superposition of RF fields from all transmit elements. This can be seen clearly with a head coil scanning a spherical phantom: when transmitting in the circularly polarised (CP+) mode, all channels have similar phase at the centre of the phantom and interfere constructively creating a strong *central brightening*; whereas when transmitting in the CP‐ mode, there is destructive interference at the centre of the phantom that causes a central *signal void* (see Figure 1A). Consequently, accurate measurement of channel‐specific $B_{1}^{+}$ phase is as important as $B_{1}^{+}$ magnitude for parallel transmission applications.

### 2.4 Time Efficiency for Many Channels

Modern pTx arrays typically include 8 or 16 transmit elements, and very rarely even more. A naïve approach of acquiring absolute $B_{1}^{+}$ maps for each transmit channel one‐by‐one requires an acquisition time proportional to the number of transmit channels, making it impractical. Considerable effort has therefore been devoted to developing calibration methods that efficiently recover per‐channel complex $B_{1}^{+}$ maps while minimising scan time and preserving SNR.

The design of the transmit array also influences the requirements for $B_{1}^{+}$ mapping. Modern pTx systems employ a variety of transmit array geometries, including loop, dipole, stripline and hybrid loop‐dipole configurations, each producing distinct transmit field distributions and coupling characteristics [28]. Coil geometry strongly affects the spatial distribution, penetration depth and dynamic range of the individual channel $B_{1}^{+}$ fields [15, 28]. For example, high‐density transmit arrays and dipole‐based designs can generate highly localised transmit sensitivities with substantial spatial variation across channels, increasing the dynamic‐range requirements of $B_{1}^{+}$ mapping methods and potentially reducing measurement robustness in low‐sensitivity regions [28, 29]. Consequently, different $B_{1}^{+}$ mapping techniques may offer advantages depending on the transmit array design. Methods such as DREAM (mentioned in Section 3 below) are attractive because of their speed and scalability to large channel counts, whereas Bloch–Siegert–based approaches can provide improved dynamic range and reduced dependence on relaxation effects [29, 30]. In addition, accurate $B_{1}^{+}$ mapping plays an important role in the evaluation and validation of novel transmit arrays, where measured transmit sensitivities are frequently compared with electromagnetic simulations to assess array performance and as a proxy to infer likely SAR prediction accuracy [28, 29].

## 3 Absolute Magnitude |$B_{1}^{+}$| Mapping

We begin recapping methods for *absolute* or *magnitude* |$B_{1}^{+}$| mapping. Most of these methods were originally conceived for single transmit coils. These sequences measure a test pulse's achieved flip angle (e.g., in degrees). With knowledge of the applied pulse waveform, that can be scaled to find the magnitude of the $B_{1}^{+}$ field in physical units (e.g., μT/V_RMS_, Hz/V_RMS_ or Hz/sqrt (W_input_)). In the context of parallel transmission, they can be applied to specific RF shims (sets of per‐channel transmit amplitude and phase scaling factors) and must be combined with other scans to map the vital per‐channel $B_{1}^{+}$ *phase* (see Section 4). The notation conventions for per‐channel $B_{1}^{+}$ mapping are shown in Table 1. Figure 4 shows the sequence diagram of eight foundational $B_{1}^{+}$ mapping methods, each with a brief introduction shown below:

**TABLE 1:** Notation conventions for per‐channel $B_{1}^{+}$ mapping.

Symbol	Variant	Units	Type	Meaning
**Electromagnetic fields** (*Accessible in simulations or* via *hardware probes in phantoms, not in vivo.)*				
$\Delta B_{0}\left(r\right)=B_{0}\left(r\right)-\nu_{\mathrm{ref}}/\gamma$		T or Hz	R	Static field offset at position r, often given in frequency units, i.e., $\gamma \Delta B_{0}\left(r\right)$. $B_{0}\left(r\right)$ is the main magnetic field. $\nu_{\mathrm{ref}}$ is the scanner reference frequency.
$B_{1}\left(r\right)=\left(B_{1x},B_{1y},B_{1z}\right)$		T	PV (1 × 3)	Time‐varying net magnetic field oscillating at the Larmor frequency at position r. In MRI, $B_{1z}$ can normally be neglected.
$B_{1}^{+}\left(r\right)=B_{1x}+iB_{1y}∠:\phi_{T}\left(r\right)$		T	P	Component of time‐varying net magnetic field at position r that rotates with the Larmor precessing spins.
$\gamma B_{1}^{+}\left(r\right)$		Hz	P	As above. Note: many authors do not write γ, implying it via units.
$B_{1,k}^{+}\left(r\right)$		T	P	Contribution to $B_{1}^{+}\left(r\right)$ from $k^{\mathrm{th}}$ transmit element
$s_{k}^{\mathrm{Tx}}\left(r\right)$	$b_{k}^{+}\left(r\right)$	T/V_RMS_	P	Transmit sensitivity of $k^{\mathrm{th}}$ transmit element, i.e., $B_{1,k}^{+}\left(r\right)$ for constant 1V_RMS_ input in phase with the scanner local oscillator.
$s_{k}^{\mathrm{Rx}}\left(r\right)$	$b_{k}^{-}\left(r\right)$	T/V or a.u.	P	Receive sensitivity of $k^{\mathrm{th}}$ receive element. Scanner receivers normally use arbitrary units.
**User controlled** *(Chosen by the user, perhaps indirectly* via *the pulse sequence or scanner software.)*				
w			CV $\left(1\times N_{c}\right)$	Per‐channel weights applied for RF shimming.
$I_{\mathrm{ref}}\left(r\right)$	$∠:\phi_{\mathrm{ref}}\left(r\right)$		C	(Virtual) reference channel pixel values. Should not contain phase poles.
α,β,…			M	Nominal flip angles in $B_{1}^{+}$‐mapping sequences. Note: actual flip‐angles $\theta \left(r\right)$ may be much larger and/or much smaller in parts of the target.
**Spin‐system response** *(Parameters that model spin dynamics, which may depend on tissue properties.)*				
$\theta \left(r\right)$		°, rad	R	Net angle of nutation of magnetisation in response to RF.
$I_{k}\left(r\right)$		a.u.	C	Pixel values in single‐channel images.
$I\left(r\right)$		a.u.	C	Pixel values in images after receive channel combination.

Abbreviations: C = complex; M = nonnegative real; P = phasor, that is, complex scalar representing a sinusoid at the Larmor frequency; R = real; V = vector; ∠ = extra symbol for the phase of the preceding quantity.

**FIGURE 4:**
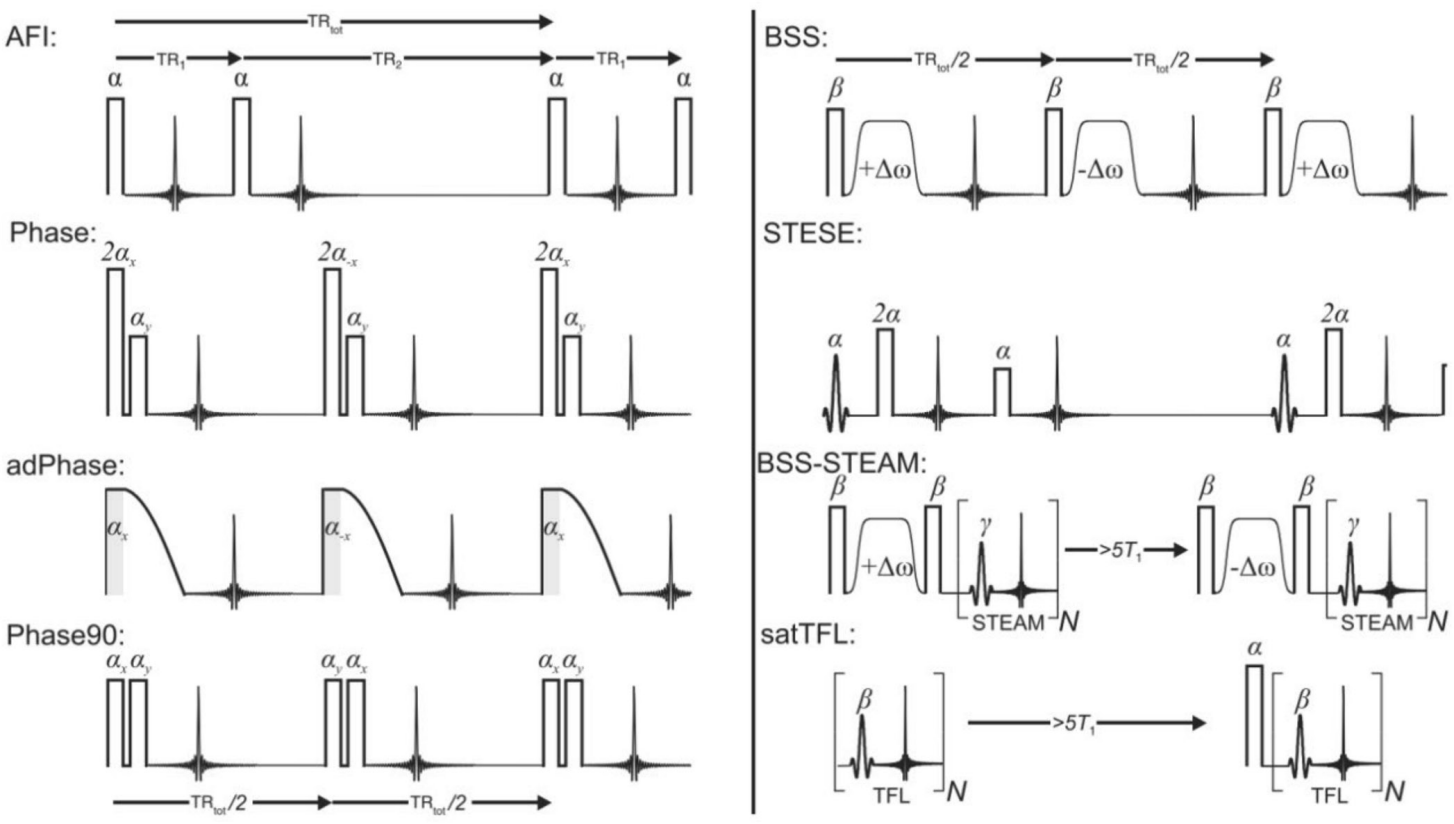
Pulse sequence diagram of eight foundational $B_{1}^{+}$ mapping methods. Sequences in the left column use 3D readouts; those in the right column use 2D readouts. Figure reproduced from [31] with permission.

### 3.1 Double‐Angle Method (DAM)

The DAM was proposed by Insko and Bolinger [32]. To apply DAM for $B_{1}^{+}$ mapping, that is, as an imaging sequence, two GRE images are typically acquired [33]. The signal intensity distribution $S_{\mathrm{GE}}\left(r\right)$ at each pixel location ***r*** is obtained from the GRE signal equations [34] assuming $\mathrm{TR}\gg T_{1}$:

(1) $$S_{\mathrm{GE}}\left(r\right)=P_{\mathrm{GE}}\left(r\right).B_{1}^{-}\left(r\right).\sin\alpha_{\mathrm{GE}}\left(r\right),$$

where $P_{\mathrm{GE}}\left(r\right)$ depends on tissue properties (proton density, T_1_ and T_2_) and imaging parameters (TE, TR and nominal flip angles); $B_{1}^{-}\left(r\right)$ is the receive sensitivity, $\alpha_{\mathrm{GE}}$ is the actual flip angle, and TR indicates the pulse's repetition time of the GRE.

Acquiring two GRE images at nominal flip angles α and 2α:

(2) $$S_{\mathrm{GE},1}\left(r\right)=P_{\mathrm{GE},1}\left(r\right).B_{1}^{-}\left(r\right).\sin\alpha \left(r\right)$$

and

(3) $$S_{\mathrm{GE},2}\left(r\right)=P_{\mathrm{GE},2}\left(r\right).B_{1}^{-}\left(r\right).\sin2\alpha \left(r\right)$$

The unknown $P_{\mathrm{GE},1}\left(r\right)$, $P_{\mathrm{GE},2}\left(r\right)$ and $B_{1}^{-}\left(r\right)$ terms are cancelled by taking the ratio, given $P_{\mathrm{GE},1}\left(r\right)=P_{\mathrm{GE},2}\left(r\right)=P_{\mathrm{GE}}\left(r\right)$ if TR > 5 T_1_, and hence estimating the flip angle as follows:

(4) $$\alpha \left(r\right)=\cos^{-1}\frac{S_{\mathrm{GE},2}\left(r\right)}{2S_{\mathrm{GE},1}\left(r\right)}$$

DAM is straightforward but needs long TRs (≥ 5T_1_) to allow full recovery before each excitation [35]. If shorter TRs are used, T_1_ effects will bias the $B_{1}^{+}$ estimate [36].

Owing to its long acquisition time, DAM is primarily of historical and educational interest and is generally not suitable for rapid pTx calibration at UHF.

### 3.2 Actual Flip‐Angle Imaging (AFI)

AFI was proposed by Yarnykh [37, 38]. It employs an interleaved dual‐TR steady‐state GRE sequence, with preparation scans to reach the driven steady state.

AFI consists of two identical RF pulses with the FA followed by two delays TR_1_ and TR_2_ (Figure 4). The FID signals S_1_ and S_2_ are observed after the corresponding pulses in a GRE form. Given the signal ratio $r=S_{2}/S_{1}$ and $n={\mathrm{TR}}_{2}/{\mathrm{TR}}_{1}$, the actual FA is estimated as follows:

(5) $$\alpha \approx \cos^{-1}\frac{\mathrm{rn}-1}{n-r}$$

AFI assumes that TR_1_ + TR_2_ ≪ T_1_. In addition, strong gradient spoiling is typically required to suppress stimulated‐echo contributions, as RF spoiling alone may not completely eliminate residual transverse magnetisation.

AFI is an accurate method with minimal T_1_ dependence, providing that the spoiler gradients are sufficient but it is limited by comparatively long scan times.

### 3.3 Saturation‐Prepared With Two Rapid Gradient Echoes (SA2RAGE)

SA2RAGE was developed by Eggenschwiler et al. [39]. SA2RAGE acquires two TurboFLASH blocks at different delay times (TD_1_ and TD_2_) after a saturation pulse.

SA2RAGE offers a wide linear dynamic range (from 0.2 to 2 times the nominal FA), low T_1_ bias and low energy deposition with relatively short scans (2 min for 3D brain $B_{1}^{+}$ map in single Tx) [40].

However, it is important to note that SA2RAGE $B_{1}^{+}$ maps exhibit significant T_1_ dependence, particularly when the T_1_ of the tissue is long relative to the timing parameters of the sequence. This is a particular problem for cerebrospinal fluid (CSF) in UHF scans [39]. To mitigate this, saturation delay and repetition time can be optimised numerically to minimise sensitivity across expected tissue T_1_ values. Sometimes SA2RAGE is post‐processed to suppress residual T_1_ effects, which improves the robustness and accuracy of the resulting $B_{1}^{+}$ maps [41].

### 3.4 Dual Refocusing Echo Acquisition Mode (DREAM)

DREAM was developed by Nehrke and Börnert for 2D multislice abdominal, head and leg $B_{1}^{+}$ mapping at 3 T [42], and further optimised by Nehrke et al. for volumetric $B_{1}^{+}$ mapping of the brain at 7 T [43]. DREAM computes the FA from the ratio of STEAM (single‐shot stimulated echo acquisition mode) and FID (free induction decay) images, acquired in a combined sequence [44].

In DREAM, the actual STEAM preparation angle α can be determined from the ratio of the STEAM signal ($S_{\mathrm{STE}}$) relative to the FID signal ($S_{\mathrm{FID}}$):

(6) $$\alpha \left(r\right)=\tan^{-1}\sqrt{2S_{\mathrm{STE}}\left(r\right)/S_{\mathrm{FID}}\left(r\right)}$$

which is sometimes presented as a normalised $B_{1}^{+}$ map:

(7) $$\mathrm{norm}\;B_{1}^{+}\left(r\right)=\alpha \left(r\right)/\alpha_{\text{nominal}}\left(r\right)\times 100\%$$

DREAM allows short acquisition times (a few seconds per slice) and does not rely on low‐bandwidth readouts (e.g., EPI or spirals) that are prone to B_0_‐induced distortions. It is less SAR‐demanding than AFI. DREAM is relatively robust to motion, exemplified by successes for measuring respiration‐induced $B_{1}^{+}$ changes in the body at 3 T [45]. However, DREAM is very sensitive to flow‐induced artefacts [46], and has a limited dynamic range of ~10°–70° [42, 47]. Furthermore, the errors induced by Rician noise increase when the SNR of one image approaches zero [42]. Slice profile effects can also cause systematic deviations in FA maps [43, 48].

Extensions of DREAM have reduced bias for 7‐T 2D neuroimaging (see Section 6), 7‐T 3D neuroimaging (i.e., 3DREAM, Section 5) and 7‐T liver imaging (Section 5).

### 3.5 Saturated Turbo FLASH (satTFL)

Klose proposed a *magnetisation prepared* 2D $B_{1}^{+}$ mapping method that utilises an RF preparation pulse, achieving 1‐min scan time per slice [49]. This was extended to create the satTFL sequence by Chung et al. [35].

satTFL acquires two FLASH images: a reference image (*S*_0_) and an image (*S*_sat_) preceded by a presaturation preparation pulse with nominal flip angle α (typically 60°–120°). The preparation pulse FA is computed as follows:

(8) $$\alpha =\cos^{-1}\left(\frac{S_{\mathrm{sat}}}{S_{0}}\right)$$

satTFL includes 2D and 3D versions. It is SAR‐efficient [31], flow‐insensitive, and intrinsically robust to motion. However, it requires a long TR for full T_1_ relaxation between the reference and magnetisation‐prepared acquisitions. Further optimisations lie in reducing this TR, such as through the ‘sandwich TFL’ method mentioned in Section 5.2. Notably, satTFL is used in the automated prescan adjustments on Siemens 7‐T MRIs.

### 3.6 Bloch–Siegert Shift (BSS) Imaging

BSS $B_{1}^{+}$ mapping was developed by Sacolick et al. [50]. BSS utilises a simple gradient‐echo sequence with a strong off‐resonant BSS‐sensitising pulse between excitation and readout. This slightly alters the effective resonance frequency of the spins [51]. That causes a phase shift which is proportional to $\int {\left(B_{1}^{+}\right)}^{2}\mathrm{dt}$ and inversely proportional to the frequency offset.

Cardiac BSS $B_{1}^{+}$ maps at 3 T showed good robustness to motion [52]. BSS is robust to T_1_ relaxation, TR, FA, chemical shift, magnetisation transfer (MT) and B_0_ inhomogeneity within a range reasonably expected in vivo [50]. However, accurate $B_{1}^{+}$ measurement needs high BSS pulse energy, which is limited by SAR at 7 T. This means that 7 T BSS needs comparatively long TRs [53].

Further work has optimised BSS in terms of its RF pulse design [54, 55, 56], pulse sequence modifications [57, 58, 59] and applications of more efficient readout schemes [60].

### 3.7 Bloch–Siegert Shift Imaging With STEAM Readout (BSS‐STEAM)

Besides the contributions on DREAM‐based $B_{1}^{+}$‐mapping methods, Nehrke and Börnert modified the standard BSS sequence to reduce SAR for 7 T [61]. In their case, only one BSS pulse is used to acquire each of the two required images, which is inserted between the first and second pulses, both with FAs of β. A stimulated echo acquisition mode (STEAM)–based imaging sequence is used after excitation of the second pulse, in which a train of small FA excitations (γ) is exploited to acquire all phase‐encoding steps in a short time. The same sequence is then repeated with a reversed offset frequency of the BSS pulse.

BSS‐STEAM showed substantial SAR reduction compared with standard BSS, with benefits in its robustness against chemical shifts and susceptibility artefacts due to a STEAM readout. STEAM has the potential to be extended by flow‐suppression gradients or chemical‐shift selective RF pulses.

### 3.8 Spin Echo Phase‐Sensitive (SEPS)

The Spin Echo Phase‐Sensitive (SEPS) method developed by Oh et al. utilises a compound‐refocusing pulse to encode $B_{1}^{+}$ into the image phase [62]. The sequence uses slice‐selective excitation followed by a non‐selective compound‐refocusing pulse, 90_*y*_ − 180_*x*_ − 90_*y*_, to form a spin echo [32] for the first image. For the second image, the compound‐refocusing pulse is modified to 90_*y*_ − 0_*x*_ − 90_*y*_, where 0_*x*_ means a time gap equal to the length of 180_*x*_ for the first image.

### 3.9 Phase Imaging (Phase) or Phase‐Sensitive Method (PS)

The original phase‐based $B_{1}^{+}$ mapping method, known as the ‘phase‐sensitive’ (PS) method, was introduced by Morrell [63]. In two images, it uses a global excitation with FA ±2α around the *x* axis, followed by α around the *y* axis. Subtracting the phase of the +2α and −2α images eliminates unwanted phase variations due to B_0_ inhomogeneity and susceptibility variations.

This can be used as‐is with modest B_0_ inhomogeneity, or can be corrected using a separately measured B_0_ map in more severe cases [64].

Although PS has been proved to be effective and more accurate over a large range of FAs than magnitude‐based DAM [32], it still requires undesirably long TRs.

### 3.10 Adiabatic Phase Imaging (adPhase)

adPhase was proposed by Hennel et al. [65, 66]. In adPhase, the two pulses in PS are merged, resulting in a pulse consisting of a simple block section, of which the FA is measured, followed by an inverse adiabatic half‐passage pulse, ensuring that the magnetisation ends up in the transverse plane [31]. In a second repetition of the excitation, the phase of the block is reversed (changing from $\alpha_{x}$ to $\alpha_{-x}$).

### 3.11 Orthogonal α (Phase90)

The orthogonal α method (Phase90) was developed by Chang [67]. Like the PS and adPhase methods, Phase90 uses two pulses with orthogonal phase and equal FA (i.e., $\alpha_{x}-\alpha_{y}$, in relation to $2\alpha_{x}-\alpha_{y}$ in PS and $\alpha_{x}$ in adPhase), taking advantage of a reduction in SAR. Phase90 acquires two scans, with a swap of the pulse phases between the first and second images (changing from $\alpha_{x}-\alpha_{y}$ to $\alpha_{y}-\alpha_{x}$).

### 3.12 Stimulated Echo/Spin Echo Imaging (STESE)

The STESE method was introduced by Akoka et al. It uses three RF pulses with FAs of α, 2*α* and α, yielding simultaneous acquisition of a spin echo (SE) image and a stimulated echo (STE) image [68].

Jiru and Klose modified STESE to use 3D EPI readouts [69].

## 4 Phase ∠$B_{1}^{+}$ Mapping

Absolute $B_{1}^{+}$ mapping methods usually only determine the *amplitude* of $B_{1}^{+}$. Yet the *phase* of $B_{1}^{+}$ is just as critical for successful pTx pulse design because it controls whether the per‐channel fields superpose constructively or destructively.

### 4.1 Relative $B_{1}^{+}$ Mapping

Relative $B_{1}^{+}$ mapping measures the complex transmit sensitivity $S_{c}\left(r\right)$ of each channel up to an unknown spatially varying complex scaling factor $\zeta \left(r\right)$ shared by all transmit channels. The classic approach relies on the fact that for low flip‐angle gradient‐recalled echo (GRE) images, the pixel complex intensity *I*(*r*) is directly proportional to $B_{1}^{+}$(*r*), where the unknown proportionality constant represents factors such as the receive coil sensitivity, the receive coil combination weights, flip angle, tissue relaxation times and so forth [11, 70]. Specifically, the relative coil transmit sensitivity:

(9) $$S_{c}^{\mathrm{rel}}\left(r\right)=S_{c}\left(r\right)\times \zeta \left(r\right)$$

can be obtained by measuring a series of low‐flip angle GRE images with RF transmit on one channel at a time $S_{c}^{\mathrm{rel}}\left(r\right)\approx I_{c}\left(r\right)$.

These relative $B_{1}^{+}$ maps are sufficient for simple RF shimming (static pTx) when the target is to maximise the constructive interference within an ROI. More importantly as explained below, they are a key input to obtain complex $B_{1}^{+}$ maps (see Figure 5).

**FIGURE 5:**
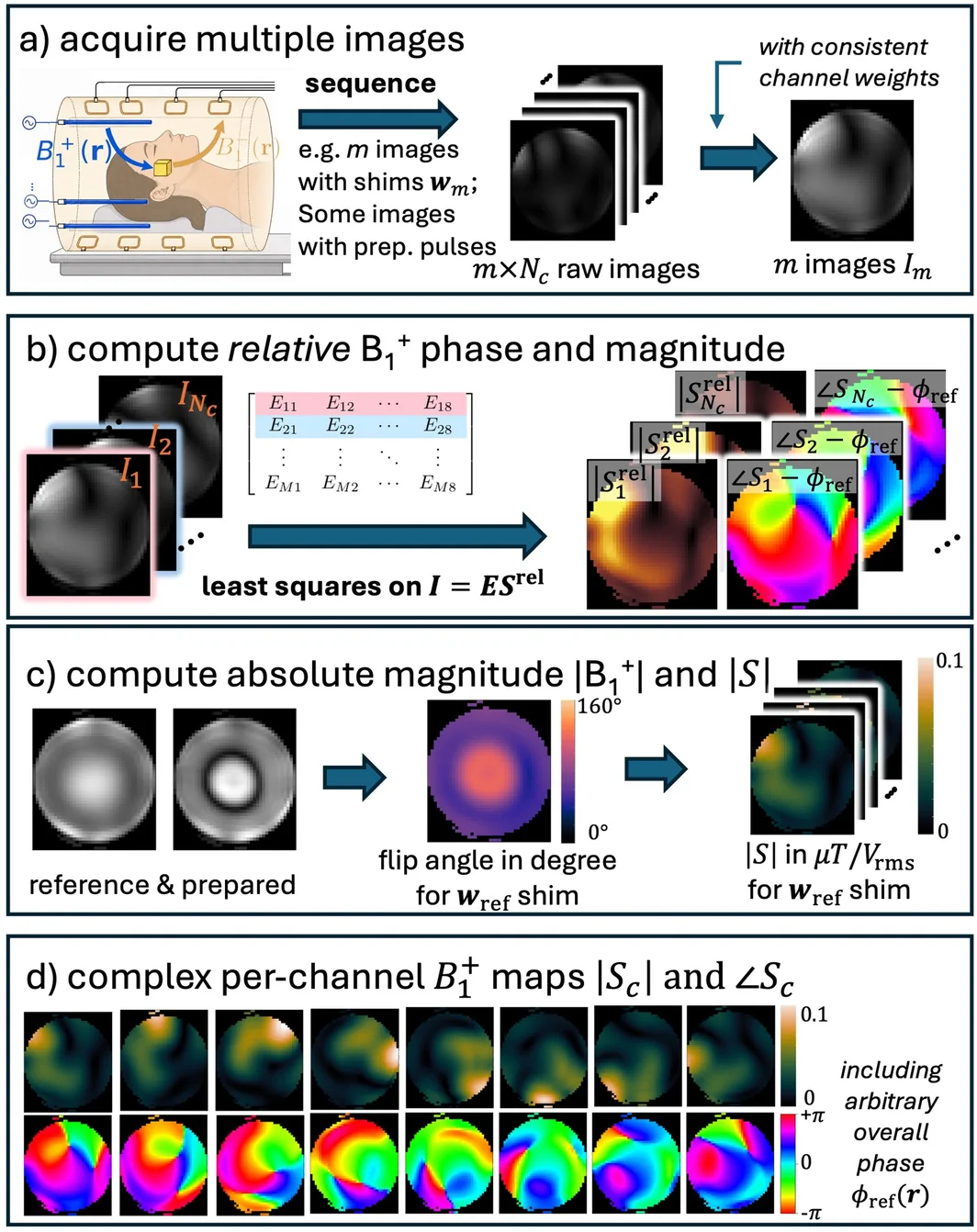
Illustration of how complex per‐channel $B_{1}^{+}$ maps are computed from a combination of relative per‐channel $B_{1}^{+}$ maps and an absolute $B_{1}^{+}$ map for one RF shim. Data shown are from a presaturated turbo‐FLASH $B_{1}^{+}$ map ‘tfl_rfmap’ acquired with the Nova 8Tx32Rx head coil on an agar phantom. (a) The coil $B_{1}^{+}$ and B_1_^−^ fields interact with the subject (or phantom) but cannot be measured directly without invasive probes. $B_{1}^{+}$ mapping methods acquire a succession of carefully chosen single channel images. (b) Relative magnitude and phase S_c_ is computed as described in Section 4 from low‐flip angle GRE images with different RF shims. (c) Absolute magnitude $B_{1}^{+}$ maps are computed as described in Section 3 in this case from the ratio of presaturated to reference image intensities, and calibrated using the known presaturation pulse to S in units of μT/V. (d) The relative and magnitude maps are combined to give complex per‐channel sensitivity maps in units of μT/V, subject to a remaining arbitrary overall phase $\phi_{\mathrm{ref}}$.

### 4.2 Interferometric Mapping

Tse et al. introduced a better *interferometric* approach for relative $B_{1}^{+}$ mapping [30]. Rather than transmitting on one channel at a time, we acquire $m\geq N_{\mathrm{cha}}$ images each with a different RF shim $w_{m}$, a $1\times N_{c}$ complex vector, which are stacked into the encoding matrix E, an $N_{m}\times N_{c}$ complex matrix. Because the RF sensitivities add

(10) $$I_{m}\left(r\right)\approx S_{m}\left(r\right)=\sum_{c}E_{\mathrm{mc}}\;S_{c}\left(r\right)$$

We solve by least squares for $\;S_{c}\left(r\right)$. Good choices of E avoid signal voids in the images $I_{m}\left(r\right)$ so that the relative phase is always well defined. Large dynamic range relative $B_{1}^{+}$ mapping approaches further improve robustness when transmit channels exhibit substantially different sensitivity profiles, which is particularly relevant for high‐channel‐count arrays and body applications [71]. For each pixel they identify which images meet the low flip‐angle criterion and trust them more in computing the final $B_{1}^{+}$ maps [71] especially for neck or body imaging [72]. Alternatively, algorithms such as rank‐deficient matrix completion can find $S_{c}\left(r\right)$ jointly for all channels [73].

### 4.3 Combining to Compute Per‐Channel Complex $B_{1}^{+}$ Maps

For large‐tip angle pTx pulse design, following van de Moortele et al. [74], we combine calibrated magnitude from absolute $B_{1}^{+}$ maps and relative phase from relative $B_{1}^{+}$ maps to give complex per‐channel $B_{1}^{+}$ maps [11, 74, 75]. This is summarised in Figure 5.

### 4.4 Overall Transmit Phase Reference

The astute reader will notice that per‐coil transmit sensitivities measured by this modern $B_{1}^{+}$ mapping approach

(11) $$S_{c}^{\text{meas}}\left(r\right)=S_{c}\left(r\right)\times \exp\left(i\;\phi_{\mathrm{ref}}\left(r\right)\right)$$

still do not completely determine the per‐channel complex transmit sensitivities $S_{c}\left(r\right)$ because they still leave an arbitrary transmit phase reference $\phi_{\mathrm{ref}}\left(r\right)$ at each pixel, as noted after eq. 10 in [76] in the context of MRS reconstruction.

Oftentimes, an arbitrary choice is made such as setting the arbitrary transmit phase reference based on the first transmit coil, that is, defining that $∠I_{1}\left(r\right)=0$ everywhere, or similarly for the CP mode RF shim. This can lead to an ambiguity between implementations.

When no single RF shim is free of signal voids everywhere, such as for body imaging, it becomes important to choose $\phi_{\mathrm{ref}}\left(r\right)$ so that it is spatially smooth and free from phase poles which can otherwise interfere with spatial smoothing steps during pTx pulse design. Promising modern approaches include ROMEO (Rapid Opensource Minimum spanning treE algorithm), MORSE‐PI and phase‐pole‐suppressed nonlinear inversion [77, 78, 79]. This problem is not yet fully solved.

## 5 Recent Advances in Rapid 3D $B_{1}^{+}$ Mapping

These challenges become increasingly important as pTx systems move towards higher channel counts and more demanding applications. In particular, robust estimation of channel‐specific complex $B_{1}^{+}$ sensitivities requires not only accurate phase referencing but also acquisition strategies that maintain reliability under realistic experimental conditions. Interleaved acquisition schemes have been proposed to improve the robustness of channel‐wise relative $B_{1}^{+}$ mapping at 7 T, while accelerated three‐dimensional multichannel $B_{1}^{+}$ mapping approaches enable more efficient characterisation of transmit sensitivities for both brain and cardiac applications [80, 81]. Such developments highlight the ongoing need for fast, reliable and scalable strategies to combine absolute field calibration with channel‐resolved complex $B_{1}^{+}$ estimation.

Recent developments in $B_{1}^{+}$ mapping have increasingly focused on volumetric (3D) acquisitions. Compared with traditional 2D approaches, 3D methods provide whole‐volume coverage, avoid slice‐to‐slice inconsistencies and generate datasets that are more suitable for pTx calibration and RF pulse design. They also facilitate retrospective slice prescription, motion‐resolved analyses and whole‐organ applications such as brain and cardiac imaging.

### 5.1 3D Free‐Breathing Absolute $B_{1}^{+}$ Mapping

Dietrich et al. developed a method for 3D respiration‐resolved absolute $B_{1}^{+}$ mapping for the human body (heart) [53]. In this approach, GRE scans were utilised to derive relative $B_{1}^{+}$ maps of the human heart, calibrated against an AFI scan. This produced absolute $B_{1}^{+}$ maps for each transmit channel in the different respiratory motion states, which were validated against conventional AFI maps for different breath‐hold positions. Unfortunately, the current implementation is rather slow requiring 3 min 24 s for GRE scans and 6 min for AFI scans, plus 15 min for motion‐binned $B_{1}^{+}$ map computation.

### 5.2 Sandwich satTFL

Kent et al. optimised satTFL to mitigate T_1_ sensitivity and reduce scan times [47]. Their ‘sandwich TFL’ method removes delays between the reference and magnetisation‐prepared images. Good agreement was found with the original (slower) satTFL method in simulations and in vivo (Figure 6). ‘Sandwich TFL’ acquires single breath‐hold whole‐brain 3D absolute $B_{1}^{+}$ maps in 20 s, and body $B_{1}^{+}$ maps in 6 min. This is competitive with 3DREAM, but does not suffer from flow sensitivity. It is possible to combine with transmit low‐rank method acceleration for even shorter scans [73].

**FIGURE 6:**
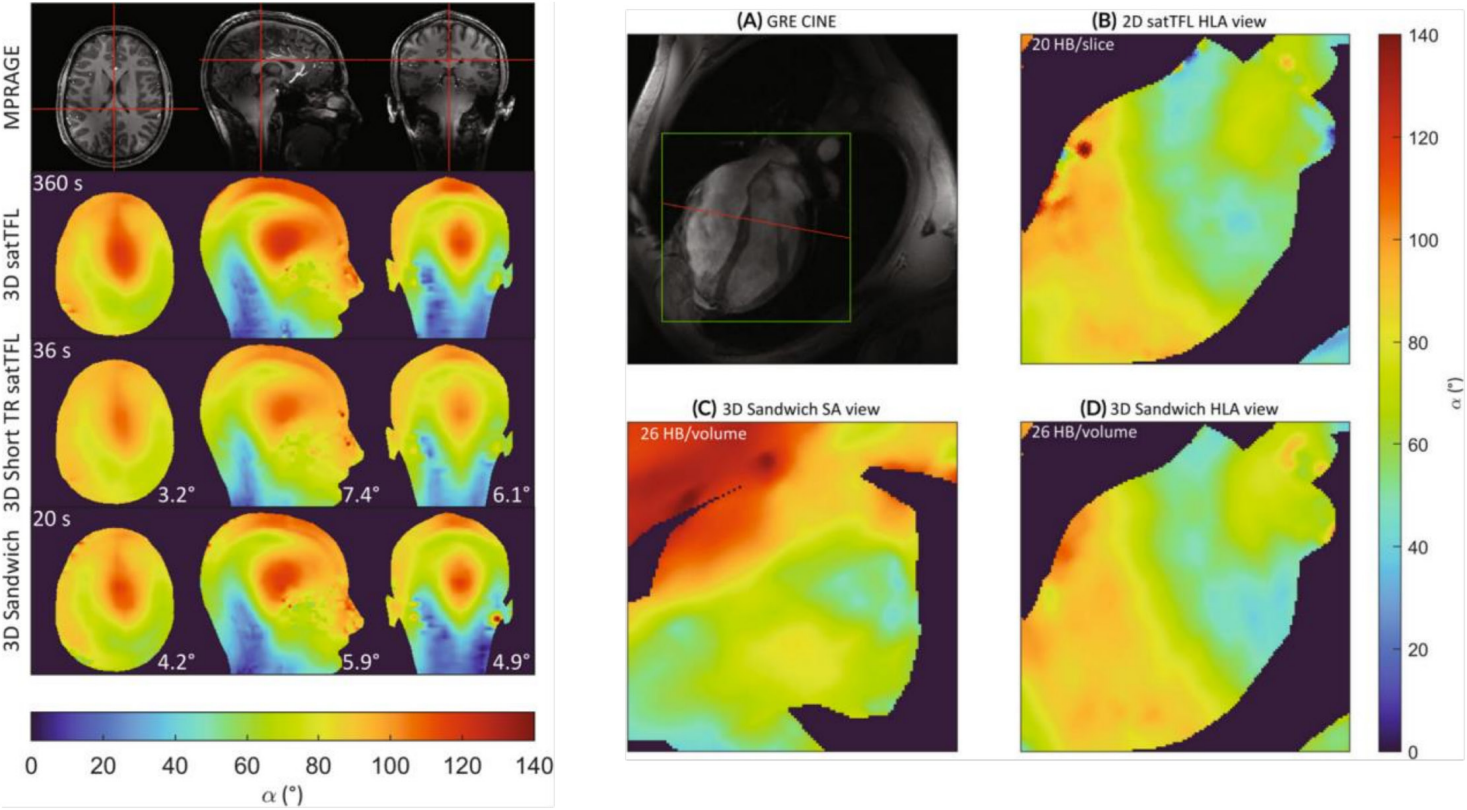
Left: 3D $B_{1}^{+}$ maps for the brain of a volunteer acquired using 3D satTFL, 3D short TR satTFL and 3D sandwich methods along with a T_1_‐weighted MPRAGE. The 3D data acquisition time (two dummy TRs included) for satTFL, short TR satTFL, and sandwich are 360, 36, and 20 s, respectively. The RMSE between map and the satTFL scheme is shown in the bottom right‐hand corner. Right: $B_{1}^{+}$ maps in the heart of a volunteer acquired using 3D sandwich sequence in CP mode. A frame from a 2D GRE CINE is shown (A) with the region of interest in green and the red line showing the short axis (SA) slice through the heart. Panel (B) is a single conventional 2D satTFL $B_{1}^{+}$ map acquired in 20 heartbeats, to be compared with 3D data acquired in 26 heartbeats (2 dummy TRs included) for sandwich along the short axis (C) and along the horizontal long axis (HLA) (D). All images acquired during breath‐hold and gated. satTFL, presaturation TurboFLASH; GRE, gradient echo. Figure reproduced from [47] with permission.

### 5.3 3DREAM

Ehses et al. introduced a 3D version of the DREAM sequence (see above) [82]. 3DREAM uses short non‐selective STE preparation and imaging pulses followed by a Cartesian centre‐out spiral phase encoded readout. This captures the desired image contrast near *k* = 0 while under‐sampling in both phase‐encoding dimensions for speed. The FID image is filtered to compensate for Gibbs ringing due to the long imaging echo train. 3DREAM produces single‐shot whole‐brain $B_{1}^{+}$ maps in a few seconds. These agree well with AFI and BSS $B_{1}^{+}$ maps (Figure 7). A pTx version acquires 8 per‐channel $B_{1}^{+}$ maps in 1 min.

**FIGURE 7:**
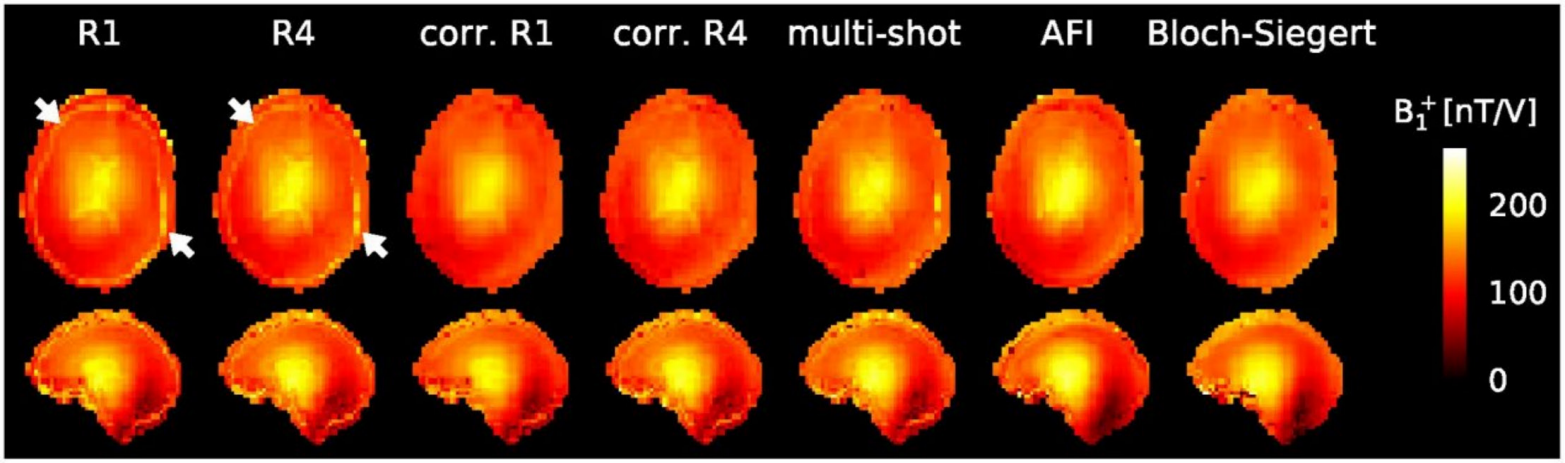
$B_{1}^{+}$ maps acquired using 3DREAM with different acceleration factors R1, R4, R1 after blurring correction (corr. R1), R4 after correction (corr. R4), multishot 3DREAM acquisition (multishot), AFI and Bloch–Siegert shift maps. Notably, ringing artefacts (denoted by white arrows) are visible around the brain boundaries in R1 and R4, and less in multishot. Figure reproduced from [82] with permission.

### 5.4 Interferometric Fourier Phase‐Encoded DREAM (PE‐DREAM)

Fourier phase‐encoded DREAM (PE‐DREAM) was developed by Tse et al. for 3D breath‐hold multichannel absolute $B_{1}^{+}$ mapping of the liver. It uses several DREAM measurements with all channels transmitting but the channel phases are cycled in unique patterns [30]. This interferometric [83] scheme improves $B_{1}^{+}$ mapping accuracy by spatially shifting regions of destructive interference to ensure accurate measurement of $B_{1}^{+}$ throughout the entire FOV, mitigating problems in regions with low SNR.

Runderkamp et al. applied PE‐DREAM for *k*_T_‐point 7‐T liver imaging [84], achieving reduced coefficients of variation (average [min‐max] across subjects) for estimated FA (7.4% [6.6%–8.0%] in *k*_T_‐points, compared with 18.8% [12.9%–23.4%], *p* < 0.001 for static phase‐only RF shimming, and 43.2% [39.4%–47.1%], *p* < 0.001 for CP mode) (Figure 8).

**FIGURE 8:**
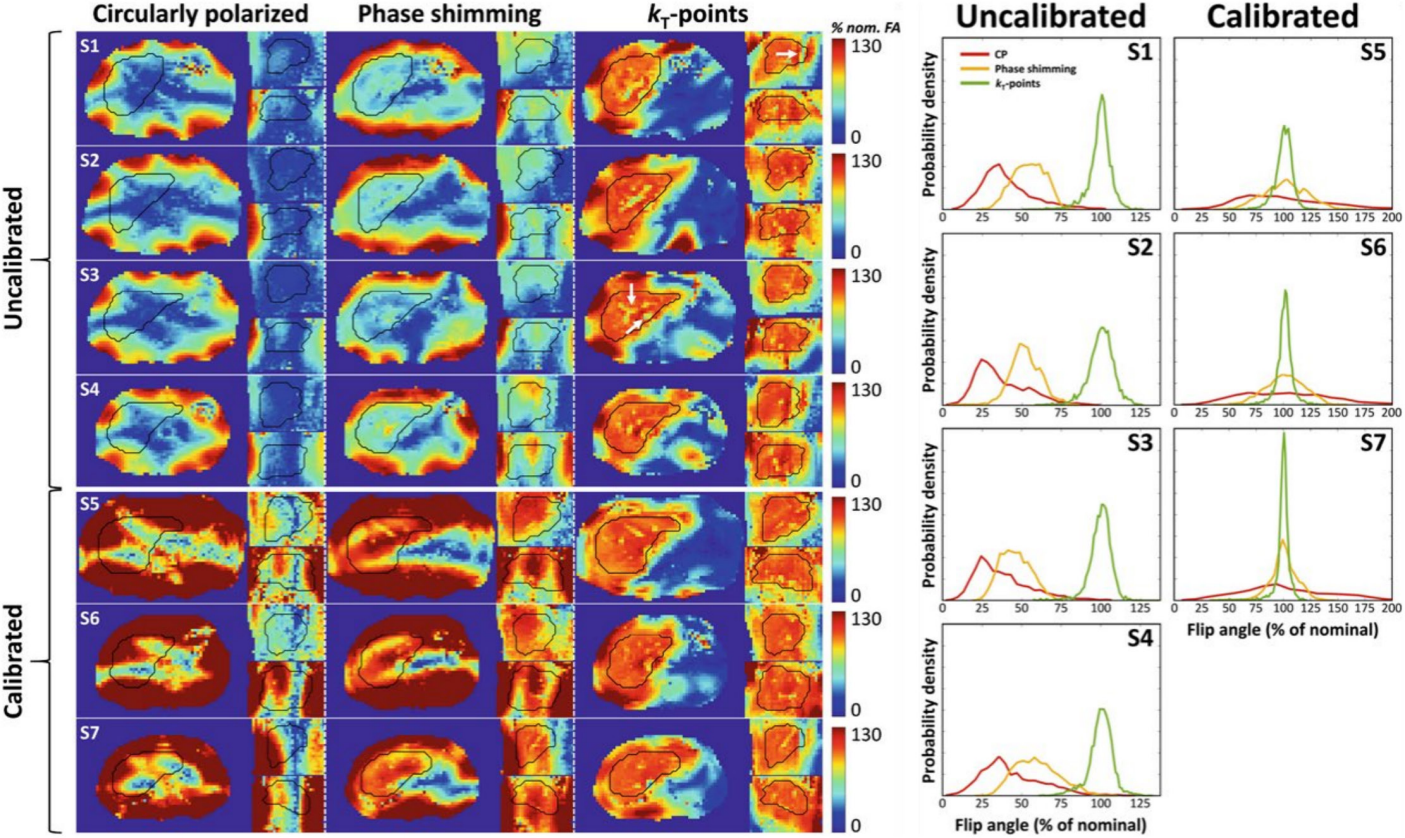
Left: Estimated FA maps for seven subjects comparing CP, phase shimming and *k*_T_‐points. Slice locations were selected centred on the largest signal dropout for CP. Black contours are the volume of interest. Subjects 1–4 were imaged with flip‐angle calibration through RF pulse area adjustment and subjects 5–7 were without calibration. The white arrows in S1 and S3 indicate example areas of residual FA inhomogeneity that are also visible in all other subjects, due to flow artefacts caused by DREAM $B_{1}^{+}$ mapping. Right: Estimated FA histograms for CP (red), phase shimming (orange) and *k*_T_‐points (green). Histograms were calculated for voxels inside the volume of interest in all 7 subjects together. Figure reproduced from [84] with permission.

### 5.5 Compressed‐Sensing Accelerated SA2RAGE (CS‐SA2RAGE)

Bonanno et al. implemented a compressed sensing readout and reconstruction for the SA2RAGE sequence (CS‐SA2RAGE) to shorten its conventional GRAPPA‐accelerated acquisition [40].

Different acceleration factors (CS) were examined against a 2 min GRAPPA reference protocol. Good agreement (< 0.05 difference) was found for most of the brain, but acquisition times were reduced up to 11‐fold. CS‐SA2RAGE enables < 30 s scan time for whole‐brain, 4‐mm isotropic resolution $B_{1}^{+}$ maps (Figure 9).

**FIGURE 9:**
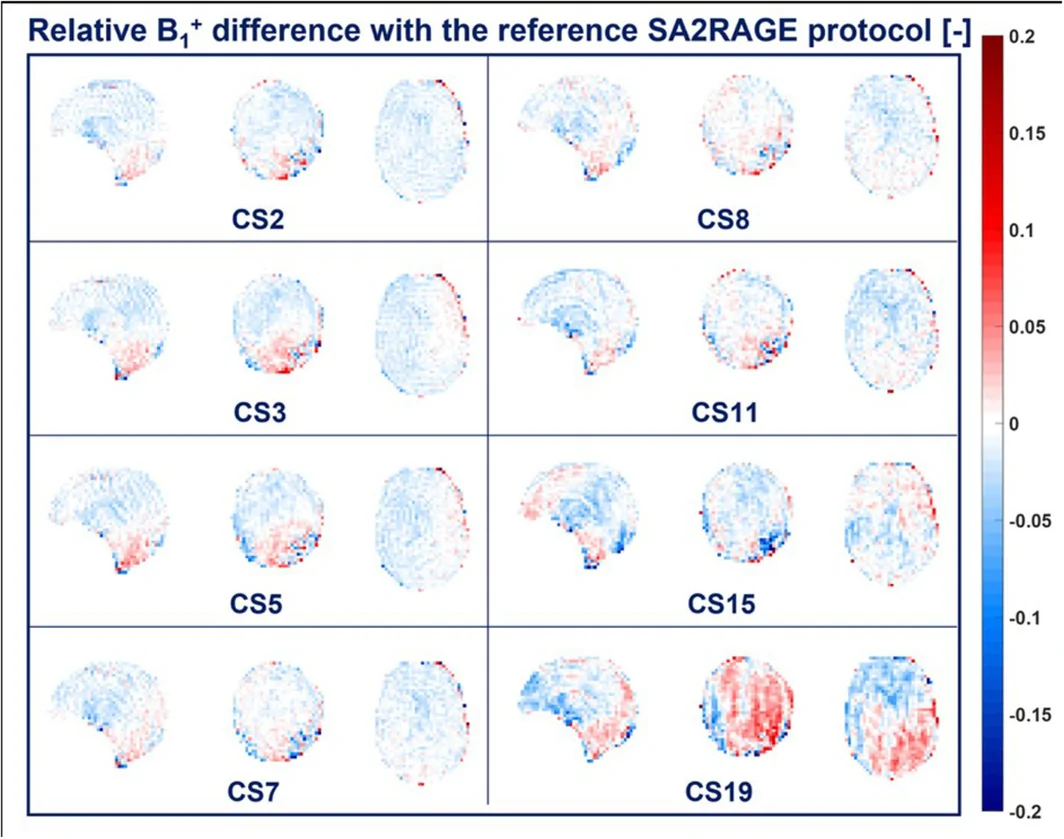
Relative $B_{1}^{+}$ difference maps of all CS‐accelerated protocols with the reference SA2RAGE protocol, shown as selected sagittal, coronal and transversal views. High acceleration factor, CS19, shows higher $B_{1}^{+}$ deviations (> ±0.05) throughout the brain. Figure reproduced from [40] with permission.

## 6 Accuracy of $B_{1}^{+}$ Maps

Knowledge of the $B_{1}^{+}$ field plays an essential role in benefiting from all the advantages that UHF offers. Accurate $B_{1}^{+}$ maps are a prerequisite to reliably optimising high‐performance pTx pulses. However, no single $B_{1}^{+}$ mapping method has yet become a universally accepted ‘gold standard’. Rather they each make different trade‐offs for factors such as accuracy, bias, dynamic range and robustness to T_1_, T_2_ and MT confounds.

### 6.1 Comparisons of Commonly Used $B_{1}^{+}$ Mapping Methods

Kent et al. assessed the sensitivity to T_1_, B_0_ and coherent flow for five 7‐T $B_{1}^{+}$ mapping sequences (satTFL [35], Sandwich TFL [47], SA2RAGE [39], AFI [37, 38] and 3DREAM [82]) by simulations [85]. AFI varied least with T_1_, had the largest dynamic range, but was most sensitive to flow. 3DREAM had the smallest dynamic range, the shortest acquisition time (TA = 4 s for 3DREAM, compared with 126 s for AFI, 87 s for SA2RAGE, 34 s for Sandwich, and 20 s for satTFL), and the lowest total energy deposition (0.48 J per channel for 3DREAM, compared with 33.41 for AFI, 4.78 for SA2RAGE, 4.00 for Sandwich TFL and 1.20 for satTFL), but was sensitive to flow‐induced artefacts. Sandwich was robust to B_0_ off resonance and had the lowest peak voltage requirements, making it appropriate for use in the body too.

Kent et al. further compared FLASH‐based $B_{1}^{+}$ mapping methods, including 2D satTFL, 3D SA2RAGE and 3D Sandwich by acquiring absolute $B_{1}^{+}$ maps in a human‐shaped phantom across multiple transmit voltages at 7 T. Both SA2RAGE and Sandwich showed good agreement with similar dynamic ranges of 20°–140° (vs. 20°–100° for satTFL), enabling low‐power non‐selective RF pulses and maintaining accurate measurements for low $B_{1}^{+}$ regions (see Figure 10A,B). However, Sandwich has a 60% shorter TA than SA2RAGE.

**FIGURE 10:**
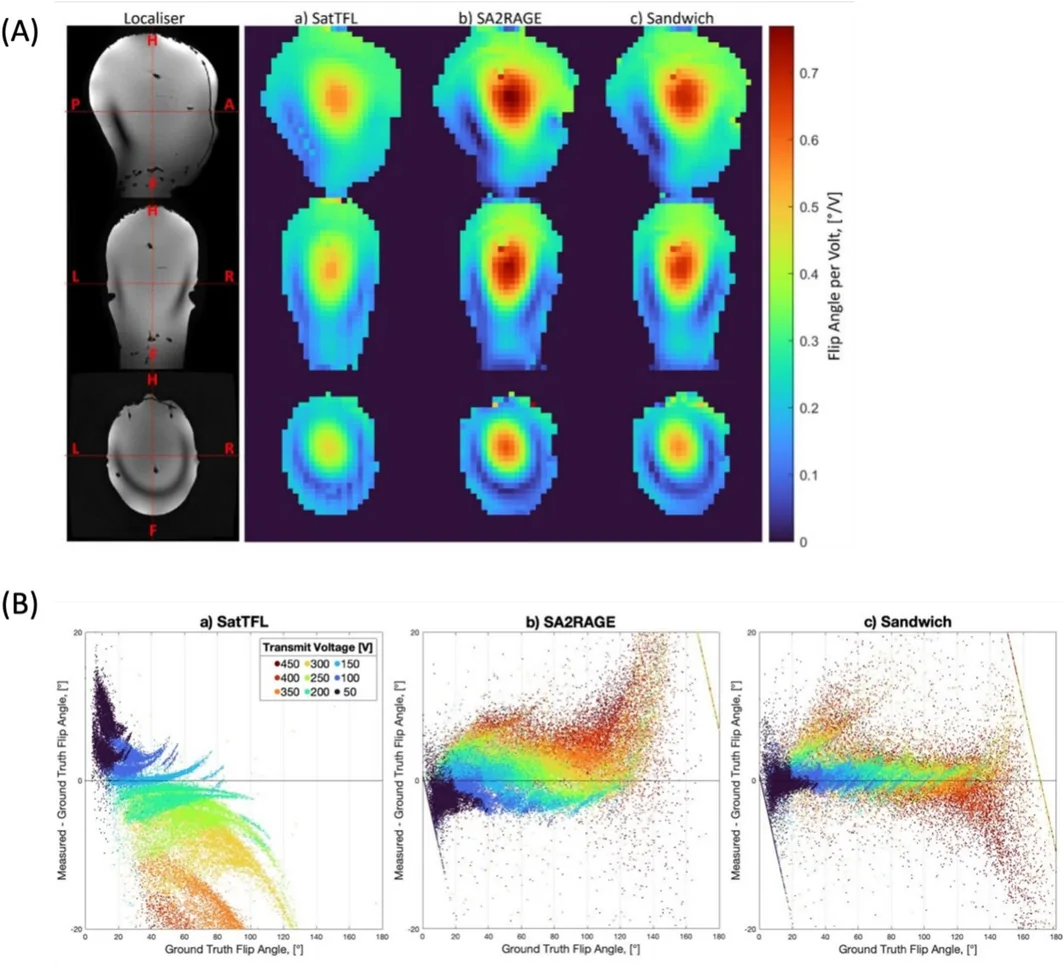
(A) Absolute $B_{1}^{+}$ maps calculated from a combination of nine transmit voltages acquired between 50 and 450 V in 50‐V increments shown in 3D view in a human head phantom. (B) Bland–Altman plots of each method, showing voxel‐wise flip‐angle measurements across the entire FOV for transmit voltages from 50 to 450 V in 50‐V increments with different colours. Sandwich and satTFL tend to underestimate high FAs, while SA2RAGE overestimates. Figure reproduced from [86] with permission.

Sedlacik et al. compared commonly used $B_{1}^{+}$ mapping methods, including AFI, SA2RAGE, satTFL (which they call ‘sat. prep. TFL’), and 3DREAM, relative to a 3D Multiple Flip Angles (MultiFA) method as reference for CP‐mode phantom scans at 7 T [87] (Figure 11A). AFI matched most closely with MultiFA, while satTFL and 3DREAM showed much lower $B_{1}^{+}$ values in the centre of the phantom (quantitative comparisons; see Figure 11B).

**FIGURE 11:**
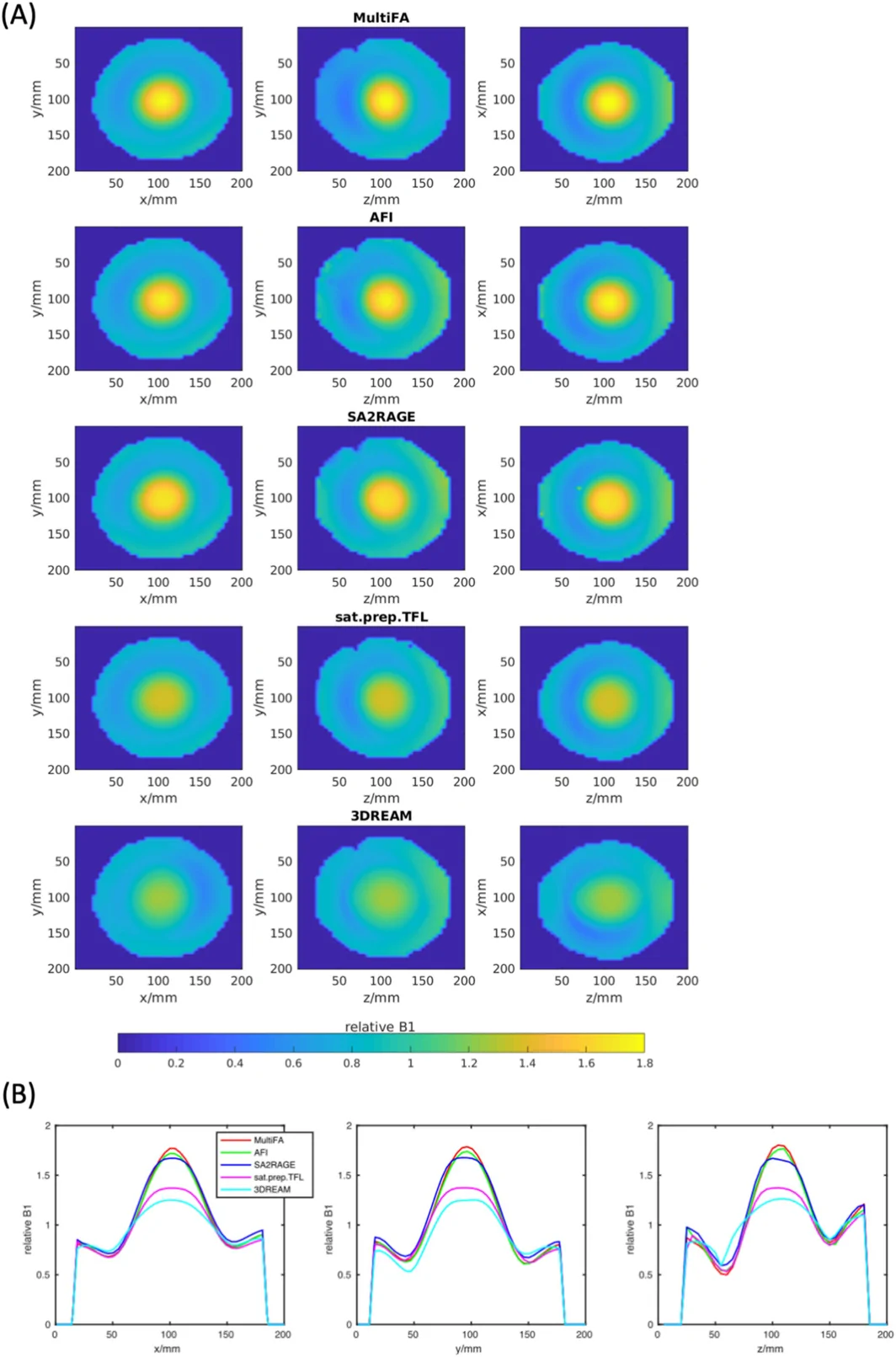
(A) Transverse, sagittal and coronal slices of the phantom $B_{1}^{+}$ maps acquired using multiFA method, AFI, SA2RAGE, sat. prep. TFL (satTFL), 3DREAM, respectively. All $B_{1}^{+}$ values are reported relative to the target $B_{1}^{+}$ value, that is, the nominal flip‐angle of each method. Note higher $B_{1}^{+}$ values in the centre of the phantom are typical for the circularly polarised (CP) mode. (B) Line‐plots of the $B_{1}^{+}$ maps through the phantom centre along the *x*, *y* and *z* directions. The $B_{1}^{+}$ values measured by AFI match well with MultiFA. Figure reproduced from [87] with permission.

### 6.2 Linear Correction for satTFL

Tomi‐Tricot et al. [87] compared satTFL $B_{1}^{+}$ values to AFI scans in 12 volunteers using an 8Tx32Rx head coil (Nova Medical Inc). They proposed that the average linear slope + intercept from the 12 participants could be applied to correct newlyacquired satTFL scans obtained with the same coil and using the same satTFL protocol (FA, TR, saturation pulse shape, etc.). Their recommended correction is as follows:

(12) $$\alpha_{\text{corrected}}=1.18\times \alpha_{\text{measured}}-0.14.$$

This correction was employed by Tomi‐Tricot et al. in their implementation of direct signal control (DSC) 2D fast spin echo (FSE) brain imaging at 7 T in the ‘DiSCoVER’ package [88]. It clearly improved image quality in vivo. This shows again the importance of *accurate* $B_{1}^{+}$ maps (Figure 12). Subsequently, the same team proposed a machine‐learning model that corrects more accurately across the whole head [89].

**FIGURE 12:**
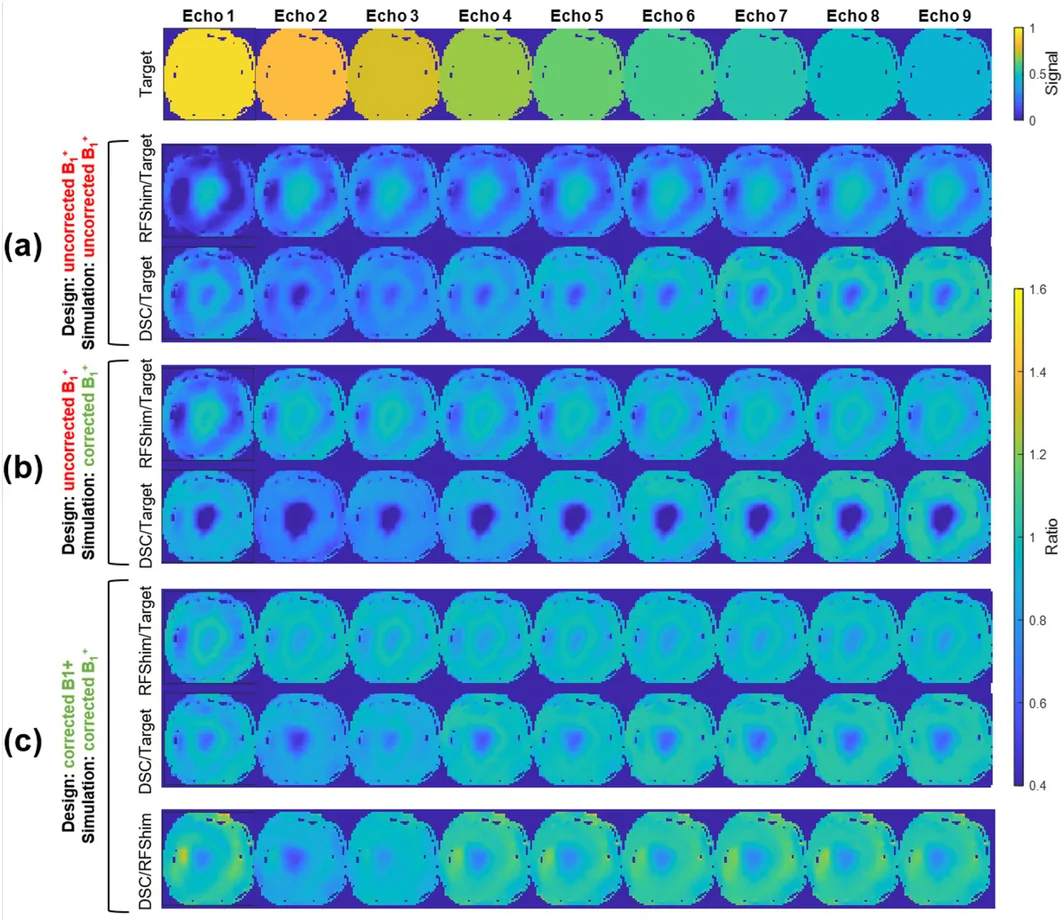
The effect of $B_{1}^{+}$ correction on target signal echoes (top row) in simulations. (a) RF shimming and DSC design and simulation conducted on uncorrected maps. (b) Same as (a) but with corrected $B_{1}^{+}$ in simulation. (c) Same as (a) but with corrected $B_{1}^{+}$ in design and simulation, along with signal ratio between DSC and RFShim. Figure reproduced from [88] with permission.

### 6.3 Bias Reduction in 2D DREAM

Olsson et al. introduced a method to increase the bias‐free $B_{1}^{+}$ range for DREAM. They acquired three separate DREAM maps with different nominal preparation FAs (25°, 40° and 60°, respectively), thresholding them to an interval of actual FA between 20° and 50° to exclude biased and low SNR pixels and compositing these into a final $B_{1}^{+}$ map [90]. Good agreement was found with AFI (Figure 13). This approach allows low‐bias, high SNR, whole‐brain $B_{1}^{+}$ maps to be acquired in < 1 min at 7 T. However, this improvement comes at the cost of increased acquisition time, which scales approximately with the number of nominal FA values acquired.

**FIGURE 13:**
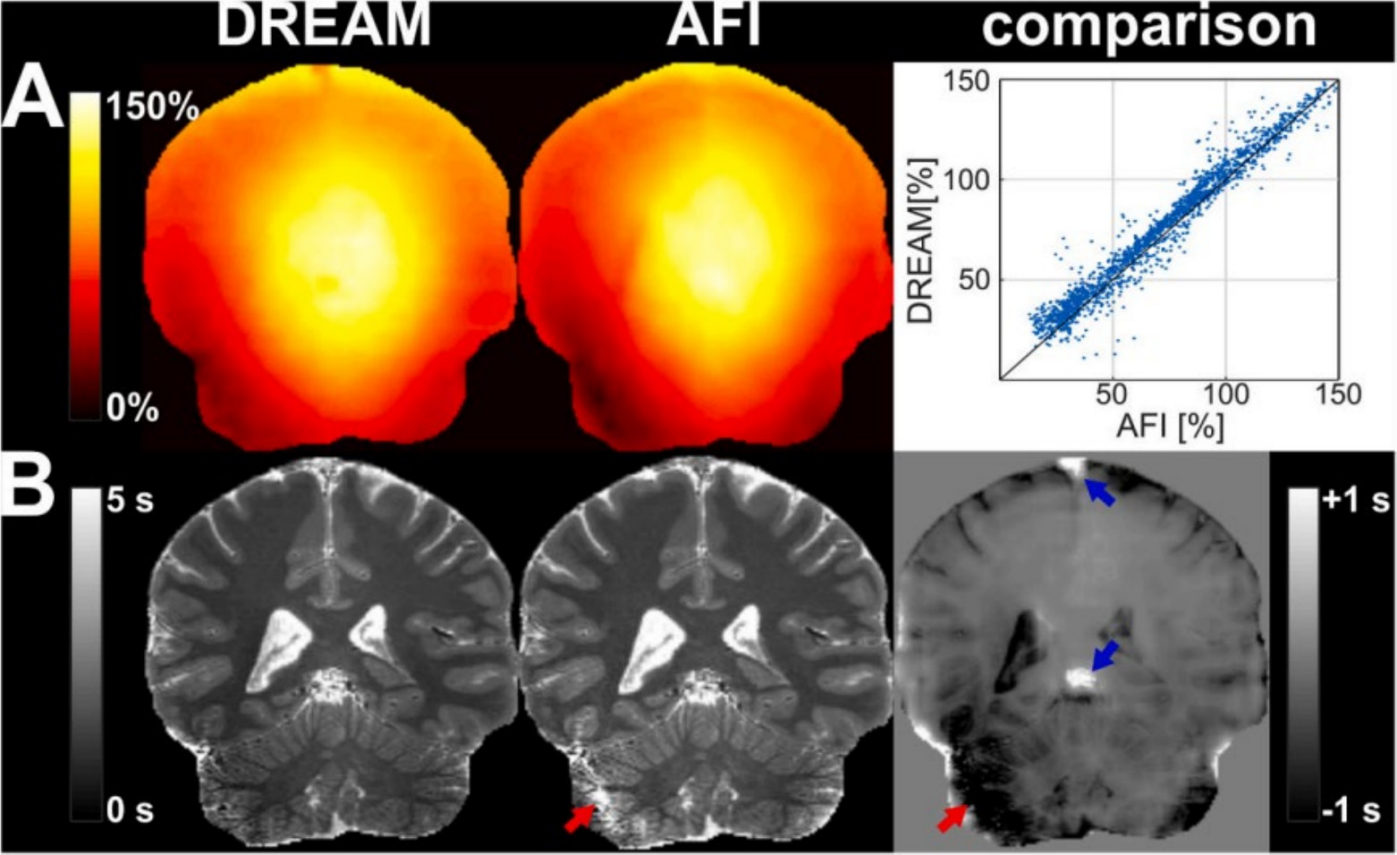
$B_{1}^{+}$ maps acquired using the combined DREAM approach or AFI (A) coregistered and interpolated to the same resolution as the T_1_ maps they are used to correct (B). A scatter plot and corresponding line of identity show the agreement between the two methods. The subtraction of the AFI‐corrected T_1_ map from the DREAM‐corrected T_1_ map is also shown. Red arrows in right cerebellum indicate lower $B_{1}^{+}$ using AFI, resulting in underestimation using DREAM and thus overestimation of T_1_ (blue arrows). Figure reproduced from [90] with permission.

## 7 Choice of $B_{1}^{+}$ Mapping Methods

In this section, the advantages and disadvantages of each $B_{1}^{+}$ mapping method are apprised to help readers select the most appropriate method for a planned study. While many $B_{1}^{+}$ mapping techniques were originally developed to measure the composite transmit field, several have subsequently been adapted for per‐channel calibration in pTx. Methods routinely extended to per‐channel $B_{1}^{+}$ mapping include AFI, BSS, DREAM, SA2RAGE, satTFL and ‘Sandwich’ satTFL. These methods are typically implemented by acquiring $B_{1}^{+}$ maps for a set of combinations of individual transmit channels, or by measuring for one mode, for example, CP and applying this to scale per‐channel relative $B_{1}^{+}$ maps. The methods included in Table 2 are presented in their per‐channel implementations, as these are the forms most relevant to pTx pulse design and RF shimming applications.

**TABLE 2:** Popular current $B_{1}^{+}$ mapping methods for human 7‐T MRI. The dynamic range, accuracy and acquisition speed ranks were based on the phantom data acquired using 7‐T Siemens Terra scanner with a 1Tx‐32Rx channel head coil in [87]. Imaging position, matrix size and field of view (voxel size = 4.5 × 4.5 × 5 mm^3^) were matched for all mapping methods of the phantom.

Name	Dimension	Type	Dynamic range rank [85]	Accuracy rank	Acquisition speed (rank) [85]	Absolute or relative	Target
Actual flip angle imaging (AFI)	3D	Magnitude	1	1	126 s (5)	Absolute	Brain and body
Bloch–Siegert shift (BSS)	2D	Phase	—	—	—	Absolute	Brain and body
Saturated turbo FLASH (satTFL)	2D	Magnitude	4	4	20 s (2)	Absolute	Brain and pelvis
Saturation‐prepared 2 rapid gradient echo (SA2RAGE)	3D	Magnitude	2	3	87 s (4)	Relative	Brain
3D Dual refocusing echo acquisition mode (3DREAM)	3D	Magnitude	5	5	4 s (1)	Absolute	Brain
Sandwich	3D	Magnitude	3	2	34 s (3)	Absolute	Brain and heart

The criteria evaluated were:

### 7.1 2D Versus 3D Method

Two‐dimensional $B_{1}^{+}$ mapping methods tend to acquire one complete *k*‐space quicker, so they are usually less sensitive to motion artefacts, which is particularly beneficial for participants who cannot keep still for extended periods (e.g., dementia patients). Two‐dimensional methods can image a single slice or a limited number of slices. Two‐dimensional methods tend to be less demanding on the system gradients. However, slice profile effects can introduce errors for 2D methods. For full volume acquisitions, 2D methods can suffer slice crosstalk and they may be slower than a 3D acquisition. Two‐dimensional methods tend to be more resistant to flow.

Three‐dimensional methods tend to have lower SAR than 2D slice‐by‐slice methods for the same coverage. They can map the entire imaging volume in a single acquisition, and they do not suffer from slice profile artefacts or slice crosstalk.

Overall, 2D schemes are favoured if only a few slices are needed, or in cases with strong motion artefacts. Three‐dimensional methods are favoured for whole‐brain scans, especially for quantitative MRI or MRSI.

### 7.2 Absolute versus Relative Method

Absolute and relative methods differ in their calculations of the $B_{1}^{+}$ (see Sections 3 and 4). In essence, absolute methods enable the exact $B_{1}^{+}$ values to be measured directly with quantitative units (e.g., in μT), while relative methods describe their spatial variations without providing exact values of $B_{1}^{+}$.

For qMRI and MRS studies (such as T_1_, T_2_/T_2_*, proton density mapping, where the measurement of signal relies on its FA that can be affected by $B_{1}^{+}$ variations, resulting in inaccurate measurement), absolute $B_{1}^{+}$ mapping methods are preferable. Relative methods can be sufficient for RF shimming or for apportioning an absolute $B_{1}^{+}$ map into per‐channel $B_{1}^{+}$ maps.

### 7.3 Magnitude‐ versus Phase‐Based Method

Magnitude‐based methods (e.g., AFI and DREAM) are sensitive to noise rectification that can overestimate $B_{1}^{+}$ in low signal areas and to saturation in areas with high $B_{1}^{+}$ or long T_1_ or T_2_ relaxation times that can underestimate $B_{1}^{+}$. Phase‐based methods (e.g., BSS and phase‐sensitive method [63]) are less affected by T_1_, T_2_ and TR (saturation effects). They tend to be less biased (but still noisy) in low signal areas. Phase‐based methods can need care to avoid or compensate for phase wrap artefacts in high $B_{1}^{+}$ areas.

### 7.4 Dynamic Range

The dynamic range of a $B_{1}^{+}$ mapping method reflects the range of $B_{1}^{+}$ values over which it can make an accurate measurement. A higher dynamic range enables the mapping method to measure low and high $B_{1}^{+}$ values accurately, which is especially important at 7 T where $B_{1}^{+}$ inhomogeneities are more pronounced. Limited dynamic range can lead to loss of mapping fidelity when measuring extreme $B_{1}^{+}$ values.

### 7.5 Compositing Multiple $B_{1}^{+}$ Maps

pTx applications in the body are challenging, partly because no single transmit element generates signal across the body which is larger than the human head.

This makes it more important to have a robust algorithm to determine the pTx $B_{1}^{+}$ map ‘gauge’ whereas in the head, simple approaches like fixing relative to the phase of CP‐mode images work acceptably. Modern approaches include VCC‐ESPIRIT, NLINV and in a recent preprint MORSE‐PI [77].

pTx scans in the body are also challenged by the dynamic range for absolute $B_{1}^{+}$ mapping. Several authors have proposed methods that acquire multiple absolute $B_{1}^{+}$ mapping scans with different pulse voltages, and that then select an appropriate combination for each pixel. So pixels near the surface will mainly be $B_{1}^{+}$ mapped using a different voltage from that used for central body pixels.

## 8 Avoiding the Need for Per‐Subject $B_{1}^{+}$ Maps

### 8.1 $B_{1}^{+}$ Map Synthesis With Generative Deep Learning (DL)

Subject‐specific tailored pTx pulse design typically requires the acquisition of a complete set of channel‐wise $B_{1}^{+}$ maps, whereas universal pulse (UP) approaches avoid subject‐specific $B_{1}^{+}$ calibration altogether. $B_{1}^{+}$ map synthesis using generative DL provides a potential intermediate strategy between these two approaches by reducing, rather than completely eliminating, the calibration requirements. Eberhardt et al. proposed acquiring only a subset of the channel‐wise $B_{1}^{+}$ maps and using generative machine learning models to synthesise the missing $B_{1}^{+}$ information [91]. The resulting combination of measured and synthesised maps can then be used for subject‐specific pTx pulse design, thereby reducing calibration time while retaining a greater degree of subject specificity than a fully calibration‐free UP approach.

In this method, image‐to‐image translation models based on generative adversarial networks, including spatially‐adaptive (de)normalisation (SPADE) [92] and pix2pixHD [93] were used to infer the missing complex $B_{1}^{+}$ maps. The synthesised maps were combined with the acquired subset of measured maps to reconstruct a complete set of channel‐wise $B_{1}^{+}$ information, which was subsequently used as input for an MLS‐based two‐spoke pTx pulse design. The resulting optimisation simultaneously determined the channel weights and spoke locations.

This approach could substantially reduce the pulse calibration time. When half of the $B_{1}^{+}$ maps were acquired and the remaining maps were synthesised, the resulting excitation performance was comparable to that obtained using a complete set of measured calibration data while reducing the calibration time by approximately 50%. However, excitation performance deteriorated as the degree of $B_{1}^{+}$ map subsampling increased. In the investigated implementation, the best performance was obtained with a subsampling factor of two, whereas larger subsampling factors resulted in less accurate synthesised maps and reduced excitation homogeneity. Performance also depended on the image synthesis model, with pix2pixHD providing slightly better results than SPADE under the investigated conditions, as illustrated in Figure 14.

**FIGURE 14:**
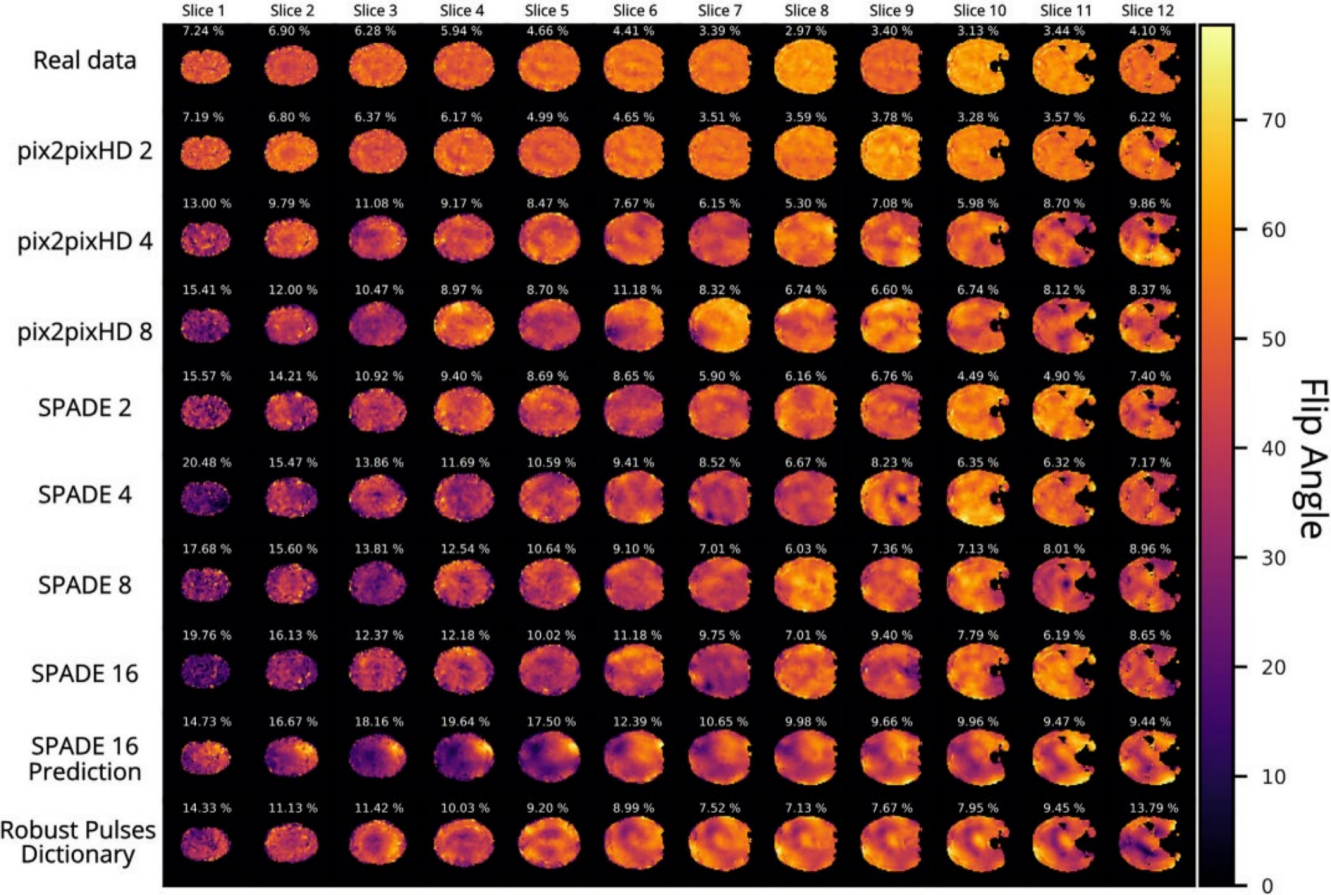
Bloch‐simulated FA maps after RF pulse optimisation based on either real $B_{1}^{+}$ data (first row) or partially subsampled $B_{1}^{+}$ data in the remaining rows. The subsampling factor is shown after the name of each generative machine learning model on the head of each row. RMSE percentage is calculated for each FA map. The last two rows show the predicted and realistic excitation patterns (taking the lowest RMSE pulse, with SPADE 16 and dictionary size 10) of the employed machine learning model for creating robust RF pulses. Figure reproduced from [91] with permission.

### 8.2 Universal pulses: A Representative Database of $B_{1}^{+}$ Maps

Universal Pulses (UPs) were introduced by Gras et al. as a calibration‐free approach to pTx pulse design. Rather than designing a pTx pulse for each individual subject, $B_{1}^{+}$ and B_0_ maps acquired from a representative group of subjects (e.g., 20 subjects in one early study) are combined into a design database, on which a generalised pTx pulse is optimised offline. The resulting pulse can then be applied to future subjects without further subject‐specific field mapping or online pulse optimisation [94]. The size and composition of the representative database will likely vary depending on factors such as the target anatomical region, transmit coil configuration, magnetic field strength and the expected participant demographics (ethnicity, BMI, age, gender, etc.). This approach can dramatically simplify the pTx workflow, usually at a cost of somewhat poorer image fidelity than fully subject‐specific pulse optimisation [95]. UPs have also been suggested to provide greater robustness to subject motion by avoiding dependence on field maps acquired at an earlier time point [96].

UPs have been applied to the key 3D non‐selective pulse sequences, such as gradient echo, MPRAGE [97], MP2RAGE [98], FLAWS (fluid and white matter suppression) [99], SPACE [100] and 3D FLAIR (FLuid Attenuated Inversion Recovery) [96] and 3D T_2_‐weighted imaging at 7 T [101] all in the PASTeUR package [102]. UPs have been refined to compensate for high B_0_ off‐resonance [103] using GRAPE [103, 104]. They have been extended to 2D and 3D reduced field of view excitations at 9.4 T [105], and the concept has been used to generate calibration‐free small region shims for single‐voxel spectroscopy [106]. More recently, group‐optimised region‐specific $B_{1}^{+}$ shims have been proposed for human brain imaging at 7 T, enabling enhancement or attenuation of $B_{1}^{+}$ within predefined regions without subject‐specific calibration [107].

Some early steps have been made to create slice‐selective UPs for GRE [108] and even with simultaneous multislice excitation [109]. This remains an open challenge for generalising UPs in 2D sequences.

### 8.3 Standardised Universal Pulses (SUP)

Standardised Universal Pulses (SUPs) were introduced by Le Ster et al. [110]. They represent a mid‐ground between the UPs and traditional per‐subject $B_{1}^{+}$ map‐based pulse designs. In SUPs, each $B_{1}^{+}$ map is normalised to a standardised database for an offline SUP design. Then for a new participant, a three‐slice rapid $B_{1}^{+}$ map is acquired in 10 s and employed to refine the SUP for that specific subject.

SUPs outperformed UPs in simulations (FA‐NRMSE of 8.8% for the UP vs. 7.1% for the adjusted SUP vs. 1.3% for the subject‐tailored pulses, on average across 15 subjects of the database), as well asand provided improved signal homogeneity and more accurate T_1_ quantification in in vivo experiments (see Figure 15, whole‐brain mean ± CV normalised FA values were 0.95 ± 0.10 for the UP, 1.00 ± 0.10 for the adjusted SUP and 0.99 ± 0.04 for tailored pulses, respectively).

**FIGURE 15:**
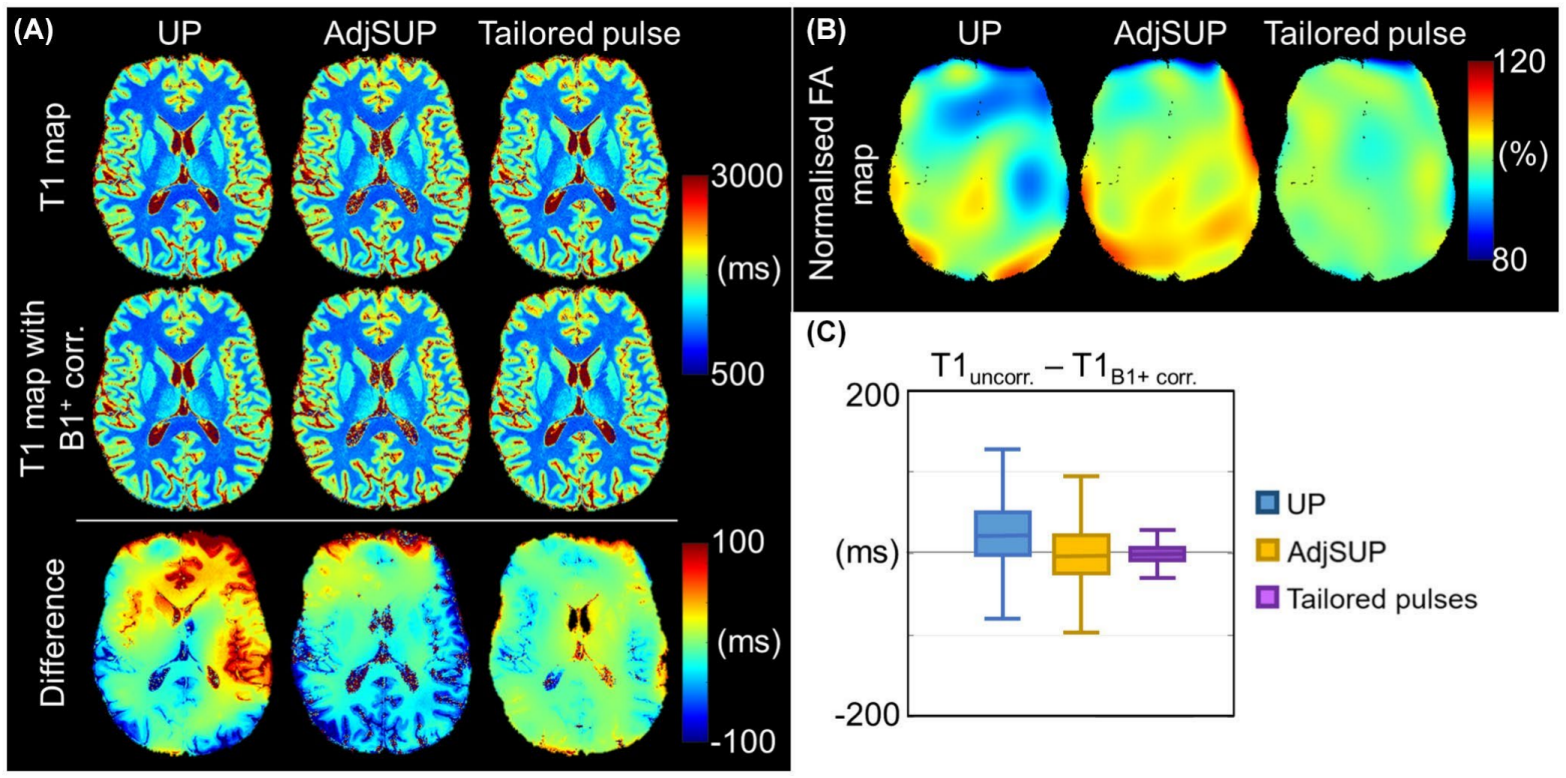
(A) T_1_ maps computed in subject 3 by injecting UNI signal intensities and sequence parameters in the Bloch simulator (numerical integration) for the MP2RAGE sequence acquired with a universal inversion pulse. From left to the right: a universal excitation pulse, an adjusted SUP excitation pulse and a subject‐tailored excitation pulse. T_1_ maps were computed without (top line) and with (middle line) $B_{1}^{+}$ correction. (B) Normalised FA maps; and (C) boxplots of the difference between T_1_ computed without and with $B_{1}^{+}$ correction. Figure reproduced from [110] with permission.

### 8.4 Fast‐Online Customised (FOCUS) Pulses

FOCUS pulses, developed by Herrler et al. [111], are another hybrid between UPs and subject‐specific pulses. Initial estimates of $B_{1}^{+}$ and B_0_ maps are made for a new subject, and used to select a *starting* pulse from a precomputed library. This is used to initialise a conventional online pulse design, in the hope of improving computation time and robustness to local minima. It has been suggested that FOCUS pulses may adapt better than UPs for patient scans, for example, postresection where there is unusual anatomy. FOCUS has also been tested for MPRAGE inversion pulses [112].

### 8.5 DL for Direct RF Pulse Design

DL methods are increasingly being used to compute pTx RF pulses directly without per‐subject $B_{1}^{+}$ maps. Examples include SmartPulse which uses a machine learning framework for calibration‐free pulse design [113] and an approach for real‐time updates of patient‐specific pTx pulses in response to motion [114].

### 8.6 BOGAT (Bayesian Optimisation of GrAdient Trajectory)

Global optimisation using BOGAT for single‐slice and simultaneous multislice imaging [115] exploits recurring patterns in $B_{1}^{+}$ maps that reflect the fixed coil geometry and similarities between human heads.

## 9 Conclusions

Parallel transmit (pTx) mitigates $B_{1}^{+}$ artefacts and improves RF safety management, helping to realise the clinical and research potential of ultra‐high‐field MRI. As field strengths continue to increase beyond 7 T, the need for accurate, robust and efficient $B_{1}^{+}$ characterisation will become increasingly pressing.

- Although recent developments such as UPs have increasingly focused on reducing the need for subject‐specific calibration, the best image quality still requires per‐subject $B_{1}^{+}$ maps.
- Yet there is an unmet need for greater harmonisation of $B_{1}^{+}$ mapping methodologies, data sharing standards and cross‐site validation procedures. We hope that this review will encourage community efforts to provide these key enablers for interoperable pTx workflows, perhaps leveraging open‐source, vendor‐independent sequence frameworks such as Pulseq. This would be an important step to support the use of parallel transmit MRI methods in multicentre translational clinical studies.

## Author Contributions

**Ye Yuan:** conceptualisation, investigation, literature review, writing – original draft, writing – review and editing, preparation of the Supporting Information and preparation of the Author Response Letter. **Jianxiang Chen:** investigation, literature review, writing – review and editing and figure preparation, including Figures 3 and 5. **Christopher T. Rodgers:** supervision, conceptualisation, writing – review and editing, manuscript submission and revision support, figure preparation and visualisation and figure caption preparation. He also provided overall project support and guidance. All authors contributed to the preparation and revision of the manuscript, reviewed the final version and approved the final manuscript.

## Funding

Christopher T. Rodgers was supported by the NIHR Cambridge Biomedical Research Centre (NIHR203312) and the Medical Research Council (UKRI226, UKRI790). The views expressed are those of the author(s) and not necessarily those of the NIHR, the NHS or the Department of Health and Social Care. For the purpose of open access, the author has applied a Creative Commons Attribution (CC BY) licence to any Author Accepted Manuscript version arising from this submission.

## Conflicts of Interest

The authors declare no conflicts of interest.

## Supporting information


**Table S1:** Currently available open‐source parallel transmit software.

## Data Availability

The data supporting the findings presented in this review are available from the corresponding author upon reasonable request. Data underlying the figures generated from experiments conducted in the authors' laboratory are available upon reasonable request.

## References

[1] B. Ding , I. Dragonu , C. Rua , et al., “Parallel Transmit (pTx) With Online Pulse Design for Task‐Based fMRI at 7 T,” Magnetic Resonance Imaging 93 (2022): 163–174, 10.1016/j.mri.2022.07.003. 35863691

[2] P.‐M. L. Robitaille , A. M. Abduljalil , A. Kangarlu , et al., “Human Magnetic Resonance Imaging at 8 T,” NMR in Biomedicine 11 (1998): 263–265, 10.1002/(SICI)1099-1492(199810)11:6<263::AID-NBM549>3.0.CO;2-0. 9802467

[3] K. Uğurbil , “Imaging at Ultrahigh Magnetic Fields: History, Challenges, and Solutions,” NeuroImage 168 (2018): 7–32, 10.1016/j.neuroimage.2017.07.007. PMC5758441 28698108

[4] S. Kashyap , D. Ivanov , M. Havlicek , S. Sengupta , B. A. Poser , and K. Uludağ , “Resolving Laminar Activation in Human V1 Using Ultra‐High Spatial Resolution fMRI at 7T,” Scientific Reports 8 (2018): 17063, 10.1038/s41598-018-35333-3. PMC6244001 30459391

[5] F. De Martino , E. Yacoub , V. Kemper , et al., “The Impact of Ultra‐High Field MRI on Cognitive and Computational Neuroimaging,” NeuroImage 168 (2018): 366–382, 10.1016/j.neuroimage.2017.03.060. 28396293

[6] A. De Ciantis , C. Barba , L. Tassi , et al., “7T MRI in Focal Epilepsy With Unrevealing Conventional Field Strength Imaging,” Epilepsia 57 (2016): 445–454. 10.1111/epi.13313 26778405

[7] D. M. Harrison , P. Sati , E. C. Klawiter , et al., “The Use of 7T MRI in Multiple Sclerosis: Review and Consensus Statement From the North American Imaging in Multiple Sclerosis Cooperative,” Brain Communications 6 (2024): fcae359, 10.1093/braincomms/fcae359. PMC11497623 39445084

[8] A. G. van der Kolk , J. Hendrikse , J. J. Zwanenburg , F. Visser , and P. R. Luijten . “Clinical Applications of 7T MRI in the Brain,” European Journal of Radiology 82 (2013): 708–718. 10.1016/j.ejrad.2011.07.007 21937178

[9] E. De Vries , C. Hagbohm , R. Ouellette , and T. Granberg , “Clinical 7 Tesla Magnetic Resonance Imaging: Impact and Patient Value in Neurological Disorders,” Journal of Internal Medicine 297 (2025): 244–261, 10.1111/joim.20059. PMC11846079 39775908

[10] J. T. Vaughan , M. Garwood , C. M. Collins , et al., “7T vs. 4T: RF Power, Homogeneity, and Signal‐to‐Noise Comparison in Head Images,” Magnetic Resonance in Medicine 46 (2001): 24–30, 10.1002/mrm.1156. 11443707

[11] P.‐F. Van de Moortele , C. Akgun , G. Adriany , et al., “B_1_ Destructive Interferences and Spatial Phase Patterns at 7 T With a Head Transceiver Array Coil,” Magnetic Resonance in Medicine 54 (2005): 1503–1518. 10.1002/mrm.20708 16270333

[12] D. I. Hoult , “Sensitivity and Power Deposition in a High‐Field Imaging Experiment,” Journal of Magnetic Resonance Imaging 12 (2000): 46–67, 10.1002/1522-2586(200007)12:1<46::AID-JMRI6>3.0.CO;2-D. 10931564

[13] C. M. Collins and M. B. Smith , “Signal‐to‐Noise Ratio and Absorbed Power as Functions of Main Magnetic Field Strength, and Definition of “90°” RF Pulse for the Head in the Birdcage Coil,” Magnetic Resonance in Medicine 45 (2001): 684–691, 10.1002/mrm.1091. 11283997

[14] M. E. Ladd , P. Bachert , M. Meyerspeer , et al., “Pros and Cons of Ultra‐High‐Field MRI/MRS for Human Application,” Progress in Nuclear Magnetic Resonance Spectroscopy 109 (2018): 1–50, 10.1016/j.pnmrs.2018.06.001. 30527132

[15] F. Padormo , A. Beqiri , J. V. Hajnal , and S. J. Malik , “Parallel Transmission for Ultrahigh‐Field Imaging: Parallel Transmission for Ultrahigh‐Field Imaging,” NMR in Biomedicine 29 (2016): 1145–1161, 10.1002/nbm.3313. PMC4995736 25989904

[16] P. M. Joseph and D. Lu , “A Technique for Double Resonant Operation of Birdcage Imaging Coils,” IEEE Transactions on Medical Imaging 8 (1989): 286–294, 10.1109/42.34719. 18230528

[17] J. Tropp , “The Theory of the Bird‐Cage Resonator,” Journal of Magnetic Resonance 2364, no. 89 (1989): 51–62, 10.1016/0022-2364(89)90164-9.

[18] T. S. Ibrahim , R. Lee , B. A. Baertlein , A. Kangarlu , and P. M. L. Robitaille , “Application of Finite Difference Time Domain Method for the Design of Birdcage RF Head Coils Using Multi‐Port Excitations,” Magnetic Resonance Imaging 18 (2000): 733–742, 10.1016/S0730-725X(00)00143-0. 10930783

[19] U. Katscher , P. Börnert , C. Leussler , and J. S. Van Den Brink , “Transmit SENSE: Transmit SENSE,” Magnetic Resonance in Medicine 49 (2003): 144–150, 10.1002/mrm.10353. 12509830

[20] I. Graesslin , P. Vernickel , P. Börnert , et al., “Comprehensive RF Safety Concept for Parallel Transmission MR,” Magnetic Resonance in Medicine 74 (2015): 589–598, 10.1002/mrm.25425. 25154815

[21] A. G. Webb and C. M. Collins , “Parallel Transmit and Receive Technology in High‐Field Magnetic Resonance Neuroimaging,” International Journal of Imaging Systems and Technology 20 (2010): 2–13, 10.1002/ima.20219.

[22] G. Adriany , P.‐F. Van De Moortele , F. Wiesinger , et al., “Transmit and Receive Transmission Line Arrays for 7 Tesla Parallel Imaging,” Magnetic Resonance in Medicine 53 (2005): 434–445, 10.1002/mrm.20321. 15678527

[23] G. J. Metzger , E. J. Auerbach , C. Akgun , et al., “Dynamically Applied *B* _1_ ^+^ Shimming Solutions for Non‐Contrast Enhanced Renal Angiography at 7.0 Tesla,” Magnetic Resonance in Medicine 69 (2013): 114–126, 10.1002/mrm.24237. PMC3387512 22442056

[24] T. Vaughan , L. DelaBarre , C. Snyder , et al., “9.4T Human MRI: Preliminary Results,” Magnetic Resonance in Medicine 56 (2006): 1274–1282, 10.1002/mrm.21073. PMC4406343 17075852

[25] D. Y. Belinda , Parallel Transmit Pulse Sequence Design for Ultra‐High Field (7T) MRI, 10.17863/CAM.96317.

[26] S. N. Williams , P. McElhinney , and S. Gunamony , “Ultra‐High Field MRI: Parallel‐Transmit Arrays and RF Pulse Design,” Physics in Medicine and Biology 68 (2023): 02TR02, 10.1088/1361-6560/aca4b7. 36410046

[27] T. H. M. Roos , E. Versteeg , M. Gosselink , et al., “pTx‐Pulseq in Hybrid Sequences: Accessible and Advanced Hybrid Open‐Source mri Sequences on Philips Scanners,” Magnetic Resonance in Medicine 94 (2025): 1946–1962, 10.1002/mrm.30601. PMC12393203 40605822

[28] C.‐H. Choi , A. Webb , S. Orzada , M. Kelenjeridze , N. J. Shah , and J. Felder , “A Review of Parallel Transmit Arrays for Ultra‐High Field MR Imaging,” IEEE Reviews in Biomedical Engineering 17 (2023): 351–368, 10.1109/RBME.2023.3244132. 37022919

[29] J. Felder , M. Zimmermann , and N. J. Shah , “High Dynamic Range $B_{1}^{+}$ Mapping for the Evaluation of Parallel Transmit Arrays,” Magnetic Resonance in Medicine 93 (2025): 1298–1305, 10.1002/mrm.30349. PMC11680734 39462464

[30] D. H. Y. Tse , M. S. Poole , A. W. Magill , J. Felder , D. Brenner , and N. Jon Shah , “Encoding Methods for $B_{1}^{+}$ Mapping in Parallel Transmit Systems at Ultra High Field,” Journal of Magnetic Resonance 245 (2014): 125–132, 10.1016/j.jmr.2014.06.006. 25036294

[31] R. Pohmann and K. Scheffler , “A Theoretical and Experimental Comparison of Different Techniques for B_1_ Mapping at Very High Fields: B_1_ Mapping Techniques,” NMR in Biomedicine 26 (2013): 265–275, 10.1002/nbm.2844. 22972684

[32] E. K. Insko and L. Bolinger , “Mapping of the Radiofrequency Field,” Journal of Magnetic Resonance, Series A 103 (1993): 82–85, 10.1006/jmra.1993.1133.

[33] V. Hartwig , N. Vanello , G. Giovannetti , et al., “ $B_{1}^{+}$/Actual Flip Angle and Reception Sensitivity Mapping Methods: Simulation and Comparison,” Magnetic Resonance Imaging 29 (2011): 717–722, 10.1016/j.mri.2011.01.004. 21524872

[34] J. Wang , M. Qiu , Q. X. Yang , M. B. Smith , and R. T. Constable , “Measurement and Correction of Transmitter and Receiver Induced Nonuniformities In Vivo,” Magnetic Resonance in Medicine 53 (2005): 408–417, 10.1002/mrm.20354. 15678526

[35] S. Chung , D. Kim , E. Breton , and L. Axel , “Rapid $B_{1}^{+}$ Mapping Using a Preconditioning RF Pulse With TurboFLASH Readout,” Magnetic Resonance in Medicine 64 (2010): 439–446, 10.1002/mrm.22423. PMC2929762 20665788

[36] D. J. Park , “B_1_ Mapping for Magnetic Resonance Imaging Brigham Young University,” (2014), 5750.

[37] V. L. Yarnykh , “Actual Flip‐Angle Imaging in the Pulsed Steady State: A Method for Rapid Three‐Dimensional Mapping of the Transmitted Radiofrequency Field,” Magnetic Resonance in Medicine 57 (2007): 192–200, 10.1002/mrm.21120. 17191242

[38] V. L. Yarnykh , “Optimal Radiofrequency and Gradient Spoiling for Improved Accuracy of *T* _1_ and *B* _1_ Measurements Using Fast Steady‐State Techniques,” Magnetic Resonance in Medicine 63 (2010): 1610–1626, 10.1002/mrm.22394. 20512865

[39] F. Eggenschwiler , T. Kober , A. W. Magill , R. Gruetter , and J. P. Marques , “SA2RAGE: A New Sequence for Fast $B_{1}^{+}$‐Mapping,” Magnetic Resonance in Medicine 67 (2012): 1609–1619, 10.1002/mrm.23145. 22135168

[40] G. Bonanno , T. Hilbert , P. Liebig , et al., “Whole‐Brain 3D $B_{1}^{+}$ Mapping in Under 30 Seconds: Compressed‐Sensing Accelerated SA2RAGE.”

[41] A. Lutti , C. Hutton , J. Finsterbusch , G. Helms , and N. Weiskopf , “Optimization and Validation of Methods for Mapping of the Radiofrequency Transmit Field at 3T,” Magnetic Resonance in Medicine 64 (2010): 229–238, 10.1002/mrm.22421. PMC3077518 20572153

[42] K. Nehrke and P. Börnert , “DREAM—A Novel Approach for Robust, Ultrafast, Multislice B_1_ Mapping,” Magnetic Resonance in Medicine 68 (2012): 1517–1526, 10.1002/mrm.24158. 22252850

[43] K. Nehrke , M. J. Versluis , A. Webb , and P. Börnert , “Volumetric $B_{1}^{+}$ Mapping of the Brain at 7T Using DREAM,” Magnetic Resonance in Medicine 71 (2014): 246–256, 10.1002/mrm.24667. 23413095

[44] J. Frahm , K. D. Merboldt , W. Hänicke , and A. Haase , “Stimulated Echo Imaging,” Journal of Magnetic Resonance 2364, no. 85 (1985): 81–93, 10.1016/0022-2364(85)90033-2.

[45] K. Nehrke and P. Börnert , “Free‐Breathing Abdominal B_1_ Mapping at 3T Using the DREAM Approach,” 2012, https://archive.ismrm.org/2012/3356.html.

[46] T. Rincón‐Domínguez , A. Menini , A. B. Solana , et al., “Accelerated Multi‐Snapshot Free‐Breathing $B_{1}^{+}$ Mapping Based on the Dual Refocusing Echo Acquisition Mode Technique (DREAM): An Alternative to Measure RF Nonuniformity for Cardiac MRI,” Magnetic Resonance Imaging 49 (2019): 499–507, 10.1002/jmri.26234. 30635987

[47] J. L. Kent , I. Dragonu , L. Valkovič , and A. T. Hess , “Rapid 3D Absolute $B_{1}^{+}$ Mapping Using a Sandwiched Train Presaturated TurboFLASH Sequence at 7 T for the Brain and Heart,” Magnetic Resonance in Medicine 89 (2023): 964–976, 10.1002/mrm.29497. PMC10099228 36336893

[48] G. Helms , J. Finsterbusch , N. Weiskopf , and P. Dechent , “Rapid Radiofrequency Field Mapping In Vivo Using Single‐Shot STEAM MRI,” Magnetic Resonance in Medicine 60 (2008): 739–743, 10.1002/mrm.21676. PMC3077516 18727090

[49] U. Klose , “Mapping of the Radio Frequency Magnetic Field With a MR Snapshot FLASH Technique: Technical Report: Mapping of the Radio Frequency Magnetic Field,” Medical Physics 19 (1992): 1099–1104, 10.1118/1.596828. 1518473

[50] L. I. Sacolick , F. Wiesinger , I. Hancu , and M. W. Vogel , “ *B* _1_ Mapping by Bloch‐Siegert Shift,” Magnetic Resonance in Medicine 63 (2010): 1315–1322, 10.1002/mrm.22357. PMC2933656 20432302

[51] F. Bloch and A. Siegert , “Magnetic Resonance for Nonrotating Fields,” Physics Review 57 (1940): 522–527, 10.1103/PhysRev.57.522.

[52] S. Weingärtner , F. Zimmer , G. J. Metzger , K. Uğurbil , P.‐F. Van de Moortele , and M. Akçakaya , “Motion‐Robust Cardiac $B_{1}^{+}$ Mapping at 3T Using Interleaved Bloch‐Siegert Shifts: Cardiac $B_{1}^{+}$ Mapping With Interleaved Bloch‐Siegert Shifts,” Magnetic Resonance in Medicine 78 (2017): 670–677, 10.1002/mrm.26395. PMC5340643 27599782

[53] S. Dietrich , C. S. Aigner , C. Kolbitsch , et al., “3D Free‐Breathing Multichannel Absolute $B_{1}^{+}$ Mapping in the Human Body at 7T,” Magnetic Resonance in Medicine 85 (2021): 2552–2567, 10.1002/mrm.28602. 33283915

[54] M. M. Khalighi , B. K. Rutt , and A. B. Kerr , “Adiabatic RF Pulse Design for Bloch‐Siegert $B_{1}^{+}$ Mapping: Adiabatic Bloch‐Siegert Pulse Design Algorithm,” Magnetic Resonance in Medicine 70 (2013): 829–835, 10.1002/mrm.24507. PMC3796009 23041985

[55] M. Jankiewicz , J. C. Gore , and W. A. Grissom , “Improved Encoding Pulses for Bloch–Siegert $B_{1}^{+}$ Mapping,” Journal of Magnetic Resonance 226 (2013): 79–87, 10.1016/j.jmr.2012.11.004. PMC3561618 23220183

[56] M. M. Khalighi , B. K. Rutt , and A. B. Kerr , “RF Pulse Optimization for Bloch‐Siegert $B_{1}^{+}$ Mapping,” Magnetic Resonance in Medicine 68 (2012): 857–862, 10.1002/mrm.23271. PMC3297726 22144397

[57] T. C. Basse‐Lüsebrink , T. Kampf , A. Fischer , et al., “SAR‐Reduced Spin‐Echo‐Based Bloch–Siegert $B_{1}^{+}$ Mapping: BS‐SE‐BURST,” Magnetic Resonance in Medicine 68 (2012): 529–536, 10.1002/mrm.23259. 22131256

[58] T. C. Basse‐Lüsebrink , V. J. F. Sturm , T. Kampf , V. J. Sturm , G. Stoll , and P. M. Jakob , “Fast CPMG‐Based Bloch‐Siegert $B_{1}^{+}$ Mapping,” Magnetic Resonance in Medicine 67 (2012): 405–418, 10.1002/mrm.23013. 21688317

[59] V. J. F. Sturm , T. C. Basse‐Lüsebrink , T. Kampf , G. Stoll , and P. M. Jakob , “Improved Encoding Strategy for CPMG‐Based Bloch‐Siegert $B_{1}^{+}$ Mapping,” Magnetic Resonance in Medicine 68 (2012): 507–515, 10.1002/mrm.23232. 22190144

[60] M. Saranathan , M. M. Khalighi , G. H. Glover , P. Pandit , and B. K. Rutt , “Efficient Bloch‐Siegert $B_{1}^{+}$ Mapping Using Spiral and Echo‐Planar Readouts,” Magnetic Resonance in Medicine 70 (2013): 1669–1673, 10.1002/mrm.24599. PMC3657582 23401024

[61] K. Nehrke , P. Börnert , H. Di , et al., “Fast B_1_ Mapping Using a STEAM‐Based Bloch‐Siegert Preparation Pulse,” 2011, https://archive.ismrm.org/2011/4411.html.

[62] C. H. Oh , S. K. Hilal , Z. H. Cho , and I. K. Mun , “Radio Frequency Field Intensity Mapping Using a Composite Spin‐Echo Sequence,” Magnetic Resonance Imaging 8 (1990): 21–25, 10.1016/0730-725X(90)90207-I. 2325512

[63] G. R. Morrell , “A Phase‐Sensitive Method of Flip Angle Mapping,” Magnetic Resonance in Medicine 60 (2008): 889–894, 10.1002/mrm.21729. 18816809

[64] S. P. Allen , G. R. Morrell , B. Peterson , et al., “Phase‐Sensitive Sodium *B* _1_ Mapping,” Magnetic Resonance in Medicine 65 (2011): 1125–1130, 10.1002/mrm.22700. PMC3073006 21413078

[65] F. Hennel and S. Köhler , “Improved Phase‐Based Adiabatic B_1_ Mapping,” 2010, https://archive.ismrm.org/2010/0237.html.

[66] F. Hennel , S. Köhler , and M. Janich , “Phase‐Sensitive B_1_ Mapping With Adiabatic Excitation,” in Proceedings of the ISMRM, 2009, 2610, https://archive.ismrm.org/2009/2610.html.

[67] Y. V. Chang , “Rapid *B* _1_ Mapping Using Orthogonal, Equal‐Amplitude Radio‐Frequency Pulses,” Magnetic Resonance in Medicine 67 (2012): 718–723, 10.1002/mrm.23051. PMC4638182 21713982

[68] S. Akoka , F. Franconi , F. Seguin , and A. Le Pape , “Radiofrequency Map of an NMR Coil by Imaging,” Magnetic Resonance Imaging 11 (1993): 437–441, 10.1016/0730-725X(93)90078-R. 8505878

[69] F. Jiru and U. Klose , “Fast 3D Radiofrequency Field Mapping Using Echo‐Planar Imaging,” Magnetic Resonance in Medicine 56 (2006): 1375–1379, 10.1002/mrm.21083. 17089359

[70] X. Wu and J. T. Vaughan , 296 Publications, 19,571.

[71] F. Padormo , A. T. Hess , P. Aljabar , et al., “Large Dynamic Range Relative $B_{1}^{+}$ Mapping: Large Dynamic Range Relative $B_{1}^{+}$ Mapping,” Magnetic Resonance in Medicine 76 (2016): 490–499, 10.1002/mrm.25884. PMC4949544 26308375

[72] M. H. S. De Buck , J. L. Kent , P. Jezzard , and A. T. Hess , “Head‐and‐Neck Multichannel $B_{1}^{+}$ Mapping and rf Shimming of the Carotid Arteries Using a 7t Parallel‐Transmit Head Coil,” Magnetic Resonance in Medicine 91 (2024): 190–204, 10.1002/mrm.29845. PMC10962593 37794847

[73] A. T. Hess , I. Dragonu , and M. Chiew , “Accelerated Calibrationless Parallel Transmit Mapping Using Joint Transmit and Receive Low‐Rank Tensor Completion,” Magnetic Resonance in Medicine 86 (2021): 2454–2467, 10.1002/mrm.28880. PMC7611890 34196031

[74] V. D. Moortele , “Calibration Tools for RF Shim at Very High Field With Multiple Element RF Coils: From Ultra Fast Local Relative Phase to Absolute Magnitude $B_{1}^{+}$ Mapping,” Proceedings of the International Society for Magnetic Resonance in Medicine 15 (2007): 1676.

[75] K. Setsompop , V. Alagappan , B. Gagoski , et al., “Slice‐Selective RF Pulses for In Vivo $B_{1}^{+}$ Inhomogeneity Mitigation at 7 Tesla Using Parallel RF Excitation With a 16‐Element Coil,” Magnetic Resonance in Medicine 60 (2008): 1422–1432, 10.1002/mrm.21739. PMC2635025 19025908

[76] C. T. Rodgers and M. D. Robson , “Coil Combination for Receive Array Spectroscopy: Are Data‐Driven Methods Superior to Methods Using Computed Field Maps?,” Magnetic Resonance in Medicine 75 (2016): 473–487, 10.1002/mrm.25618. PMC4744755 25820303

[77] B. Dymerska , O. Josephs , N. Graedel , et al., “MORSE‐PI—Flexible and Artefact‐Free Image Reconstruction for Structural and Functional QSM and Other Phase‐Critical Imaging Applications,” (2026), Toronto, ON, Canada, 4265, https://arxiv.org/abs/2606.21336.

[78] M. Blumenthal and M. Uecker , “Phase‐Pole‐Free Images and Smooth Coil Sensitivity Maps by Regularized Nonlinear Inversion,” Magnetic Resonance in Medicine 96 (2026): 134–145, 10.1002/mrm.70333. PMC13156459 41820227

[79] B. Dymerska , K. Eckstein , B. Bachrata , et al., “Phase Unwrapping With a Rapid Opensource Minimum Spanning Tree Algorithm (ROMEO),” Magnetic Resonance in Medicine 85 (2021): 2294–2308, 10.1002/mrm.28563. PMC7821134 33104278

[80] N. Egger , L. Ruck , S. Nagelstraßer , et al., “Evaluation of an Interleaved Acquisition Scheme for Improved Robustness of Channel‐Wise Relative $B_{1}^{+}$ Mapping at 7 t ,” Magnetic Resonance in Medicine 95 (2026): 1157–1168, 10.1002/mrm.70101. PMC12681310 41024603

[81] J. L. Kent , M. H. S. De Buck , I. Dragonu , et al., “Accelerated 3d Multi‐Channel $B_{1}^{+}$ Mapping at 7 t for the Brain and Heart,” Magnetic Resonance in Medicine 92 (2024): 2007–2020, 10.1002/mrm.30201. PMC7618857 38934380

[82] P. Ehses , D. Brenner , R. Stirnberg , E. D. Pracht , and T. Stöcker , “Whole‐Brain B_1_‐Mapping Using Three‐Dimensional DREAM,” Magnetic Resonance in Medicine 82 (2019): 924–934, 10.1002/mrm.27773. 31038244

[83] D. O. Brunner and K. P. Pruessmann , “ $B_{1}^{+}$ Interferometry for the Calibration of RF Transmitter Arrays: $B_{1}^{+}$ Interferometry for Array Calibration,” Magnetic Resonance in Medicine 61 (2009): 1480–1488, 10.1002/mrm.21893. 19353666

[84] B. A. Runderkamp , T. Roos , W. Van Der Zwaag , et al., “Whole‐Liver Flip‐Angle Shimming at 7 t Using Parallel‐Transmit * k * _ t _‐Point Pulses and Fourier Phase‐Encoded dream $B_{1}^{+}$ Mapping,” Magnetic Resonance in Medicine 91 (2024): 75–90, 10.1002/mrm.29819. 37799015

[85] J. L. Kent , L. Valkovič , I. Dragonu , and A. T. Hess , “A Simulation Framework for Comparison of Volumetric $B_{1}^{+}$ Mapping Methods.”

[86] J. L. Kent and P. Liebig , “A Comparison of FLASH‐Based Volumetric $B_{1}^{+}$ Mapping Methods in Phantom at 7 Tesla.”

[87] J. Sedlacik , R. Tomi‐Tricot , T. Wilkinson , et al., “Calibration of Saturation‐Prepared Turbo FLASH $B_{1}^{+}$ Maps by Actual Flip Angle Imaging at 7T,” in Proceedings of the 30th Joint Annual ISMRM‐ESMRMB Meeting, London, UK, 2022, 2867.

[88] R. Tomi‐Tricot , J. Sedlacik , J. Endres , et al., “Fully Integrated Scanner Implementation of Direct Signal Control for 2D T2‐Weighted TSE at Ultra‐High Field,” in Proceedings of the International Society for Magnetic Resonance in Medicine, 2021, 621.

[89] M. Dubiner , J. Sedlacik , T. Wilkinson , et al., “A Machine‐Learning Approach for Saturation‐Prepared Turbo FLASH $B_{1}^{+}$ Maps Calibration,” Toronto, ON, Canada, 0953.

[90] H. Olsson , M. Andersen , and G. Helms , “Reducing Bias in DREAM Flip Angle Mapping in Human Brain at 7T by Multiple Preparation Flip Angles,” Magnetic Resonance Imaging 72 (2020): 71–77, 10.1016/j.mri.2020.07.002. 32645432

[91] B. Eberhardt , B. A. Poser , N. J. Shah , and J. Felder , “B_1_ Field Map Synthesis With Generative Deep Learning Used in the Design of Parallel‐Transmit RF Pulses for Ultra‐High Field MRI,” Zeitschrift für Medizinische Physik 32 (2022): 334–345, 10.1016/j.zemedi.2021.12.003. PMC9948838 35144850

[92] T. Park , M.‐Y. Liu , T.‐C. Wang , and J.‐Y. Zhu , “Semantic Image Synthesis With Spatially‐Adaptive Normalization.”

[93] T.‐C. Wang , M.‐Y. Liu , J.‐Y. Zhu , et al., “High‐Resolution Image Synthesis and Semantic Manipulation With Conditional GANs,” in 2018 IEEE/CVF Conference on Computer Vision and Pattern Recognition (IEEE, 2018), 8798–8807.

[94] V. Gras , A. Vignaud , A. Amadon , D. le Bihan , and N. Boulant , “Universal Pulses: A New Concept for Calibration‐Free Parallel Transmission,” Magnetic Resonance in Medicine 77 (2017): 635–643, 10.1002/mrm.26148. 26888654

[95] C. S. Aigner , S. Dietrich , T. Schaeffter , and S. Schmitter , “Calibration‐Free pTx of the Human Heart at 7T via 3D Universal Pulses,” Magnetic Resonance in Medicine 87 (2022): 70–84, 10.1002/mrm.28952. 34399002

[96] V. Gras , E. D. Pracht , F. Mauconduit , D. Le Bihan , T. Stöcker , and N. Boulant , “Robust Nonadiabatic T_2_ Preparation Using Universal Parallel‐Transmit k_T_‐Point Pulses for 3D FLAIR Imaging at 7 T,” Magnetic Resonance in Medicine 81 (2019): 3202–3208, 10.1002/mrm.27645. 30652352

[97] J. P. Mugler and J. R. Brookeman , “Three‐Dimensional Magnetization‐Prepared Rapid Gradient‐Echo Imaging (3D MP RAGE),” Magnetic Resonance in Medicine 15 (1990): 152–157, 10.1002/mrm.1910150117. 2374495

[98] J. P. Marques , T. Kober , G. Krueger , W. van der Zwaag , P. F. Van de Moortele , and R. Gruetter , “MP2RAGE, a Self Bias‐Field Corrected Sequence for Improved Segmentation and T_1_‐Mapping at High Field,” NeuroImage 49 (2010): 1271–1281, 10.1016/j.neuroimage.2009.10.002. 19819338

[99] J. Beaumont , G. Gambarota , H. Saint‐Jalmes , et al., “High‐Resolution Multi‐T_1_–Weighted Contrast and T_1_ Mapping With Low $B_{1}^{+}$ Sensitivity Using the Fluid and White Matter Suppression (FLAWS) Sequence at 7T,” Magnetic Resonance in Medicine 85 (2021): 1364–1378, 10.1002/mrm.28517. 32989788

[100] J. P. Mugler , “Optimized Three‐Dimensional Fast‐Spin‐Echo MRI,” Magnetic Resonance Imaging 39 (2014): 745–767, 10.1002/jmri.24542. 24399498

[101] V. Gras , F. Mauconduit , A. Vignaud , et al., “Design of Universal Parallel‐Transmit Refocusing k_T_‐Point Pulses and Application to 3D T_2_ ‐Weighted Imaging at 7T: Universal Pulse Design of 3D Refocusing Pulses,” Magnetic Resonance in Medicine 80 (2018): 53–65, 10.1002/mrm.27001. 29193250

[102] A. Massire and M. Naudin , “PASTEUR: Package of Anatomical Sequences Using Parallel Transmission Universal Pulses Now Available for MAGNETOM Terra.”

[103] L. Van Damme , F. Mauconduit , T. Chambrion , N. Boulant , and V. Gras , “Universal Nonselective Excitation and Refocusing Pulses With Improved Robustness to Off‐Resonance for Magnetic Resonance Imaging at 7 Tesla With Parallel Transmission,” Magnetic Resonance in Medicine 85 (2021): 678–693, 10.1002/mrm.28441. 32755064

[104] N. Khaneja , T. Reiss , C. Kehlet , T. Schulte‐Herbrüggen , and S. J. Glaser , “Optimal Control of Coupled Spin Dynamics: Design of NMR Pulse Sequences by Gradient Ascent Algorithms,” Journal of Magnetic Resonance 172 (2005): 296–305, 10.1016/j.jmr.2004.11.004. 15649756

[105] O. Geldschläger , D. Bosch , S. Glaser , and A. Henning , “Local Excitation Universal Parallel Transmit Pulses at 9.4T,” Magnetic Resonance in Medicine 86 (2021): 2589–2603, 10.1002/mrm.28905. 34180089

[106] A. Berrington , M. Považan , C. Mirfin , et al., “Calibration‐Free Regional RF Shims for MRS,” Magnetic Resonance in Medicine 86 (2021): 611–624, 10.1002/mrm.28749. 33749010

[107] E. J. P. Brouwer , W. Van Der Zwaag , J. Heij , and N. Priovoulos , “Easy‐to‐Use $B_{1}^{+}$ Shims for Human Brain Imaging at 7 T,” Magnetic Resonance in Medicine 94 (2025): 2010–2022, 10.1002/mrm.30617. PMC12393206 40554723

[108] V. Gras , M. Boland , A. Vignaud , et al., “Homogeneous Non‐Selective and Slice‐Selective Parallel‐Transmit Excitations at 7 Tesla With Universal Pulses: A Validation Study on Two Commercial RF Coils,” PLoS ONE 12 (2017): e0183562, 10.1371/journal.pone.0183562. PMC5565195 28827835

[109] C. Le Ster , A. Moreno , F. Mauconduit , et al., “Comparison of SMS‐EPI and 3D‐EPI at 7T in an fMRI Localizer Study With Matched Spatiotemporal Resolution and Homogenized Excitation Profiles,” PLoS ONE 14 (2019): e0225286, 10.1371/journal.pone.0225286. PMC6872176 31751410

[110] C. Le Ster , F. Mauconduit , A. Massire , N. Boulant , and V. Gras , “Standardized Universal Pulse: A Fast RF Calibration Approach to Improve Flip Angle Accuracy in Parallel Transmission,” Magnetic Resonance in Medicine 87 (2022): 2839–2850, 10.1002/mrm.29180. 35122302

[111] J. Herrler , P. Liebig , R. Gumbrecht , et al., “Fast Online‐Customized (FOCUS) Parallel Transmission Pulses: A Combination of Universal Pulses and Individual Optimization,” Magnetic Resonance in Medicine 85 (2021): 3140–3153, 10.1002/mrm.28643. 33400302

[112] J. Herrler , S. N. Williams , P. Liebig , et al., “Evaluating Universal and Fast Online Customized Pulses for Parallel Transmission Using Two Different RF Coils,” International Society for Magnetic Resonance in Medicine, 2021, 3950.

[113] R. Tomi‐Tricot , V. Gras , B. Thirion , et al., “SmartPulse, a Machine Learning Approach for Calibration‐Free Dynamic RF Shimming: Preliminary Study in a Clinical Environment,” Magnetic Resonance in Medicine 82 (2019): 2016–2031, 10.1002/mrm.27870. 31257612

[114] E. Kopanoglu , “Patient‐Specific Parallel Transmit Pulses Are Patient Position Dependent While Safety Models Are Fixed: Safety Implications.”

[115] M. Zhang and C. T. Rodgers , “Bayesian Optimization of Gradient Trajectory for Parallel‐Transmit Pulse Design,” Magnetic Resonance in Medicine 91 (2024): 2358–2373, 10.1002/mrm.30007. 38193277

