# Supplementary material for: Per‐Channel Complex B1+ Mapping for Parallel Transmit Ultra‐High Field MRI
Source: NMR Biomed. 2026 Nov 3;39(12):e70425. doi: 10.1002/nbm.70425 (PMC13732111; doi:10.1002/nbm.70425)
Supplement: Supplementary file 1 [file NBM-39-e70425-s001.docx]

# Supplementary information for “Per-channel Complex B1+ Mapping for Parallel Transmit Ultra-high field MRI”

Ye Yuan^1^, Jianxiang Chen^1^, Christopher T. Rodgers^1^*

Submitted to NMR in Biomedicine.

Supplementary Table 1. Currently available open-source parallel transmit software.

| Lab | Package(s) | 2D | 3D |
| --- | --- | --- | --- |
| Tübingen | FastPtx ^1^: <https://github.com/dabosch/FastPtx>  OTUP ^2^:  <https://github.com/ole1965/workflow_OTUP> | Y | Y |
| Vanderbilt | k-space domain design: <https://github.com/wgrissom/kpTx/tree/master>  SigPy.RF ^3^ <https://cds.ismrm.org/protected/20MProceedings/PDFfiles/1045.html> <https://github.com/mikgroup/sigpy>  PulPy (successor) ^4^ <https://github.com/jonbmartin/pulpy> | Y | N |
| Utrecht | pTx-Pulseq ^5^  <https://openmr.nl/> | Y (plays pre-designed sequence) | Y (plays pre-designed sequence) |
| King’s College London | PUSH, HybridPTx at ^6^  <https://gitlab.com/mriphysics/> | Y | Y |
| Bonn | kT-points <https://github.com/mrphysics-bonn/interleaved_binomial_kTpoints> |  |  |
| Martinos PTx Codes | Not accessible at the time of writing ^7^:  <https://web.archive.org/web/20230315092729/https://ptx.martinos.org/index.php?title=Main_Page>  <https://github.com/john-drago/MP-pTx-STA> |  |  |
| Oxford | Low-rank B_1_^+^ mapping  <https://github.com/mchiew/txlr> |  |  |
| Minnesota | e.g. <https://github.com/XiaopingWu2020/ptx-multiband> <https://github.com/XiaopingWu2020/ptx-multispokes>  <https://github.com/Tsinghua-CBIR/pTx-SPSP> |  |  |
| Aarhus | blOCh: <https://github.com/madssakre/blOCh> |  |  |
| PTB | UP_body: <https://github.com/chaigner/UP_body> |  |  |

**References**

1.	Bosch D, Scheffler K. FastPtx: a versatile toolbox for rapid, joint design of pTx RF and gradient pulses using Pytorch’s autodifferentiation. *Magn Reson Mater Phy*. 2023;37(1):127-138. doi:10.1007/s10334-023-01134-7

2.	Geldschläger O, Bosch D, Henning A. OTUP workflow: target specific optimization of the transmit k‐space trajectory for flexible universal parallel transmit RF pulse design.

3.	Martin JB, Ong F, Ma J, Tamir JI, Lustig M, Grissom WA. SigPy. RF: comprehensive open-source rf pulse design tools for reproducible research. In: *Proceedings of the International Society for Magnetic Resonance in Medicine. ISMRM Annual Meeting*. Vol 1045. 2020.

4.	Martin JB, Sun H, Albert M, Johnson KM, Grissom WA. PulPy: A Python Toolkit for MRI RF and Gradient PulseDesign. *JOSS*. 2024;9(103):6586. doi:10.21105/joss.06586

5.	Roos THM, Versteeg E, Gosselink M, et al. pTx‐Pulseq in hybrid sequences: Accessible and advanced hybrid open‐source MRI sequences on Philips scanners. *Magnetic Resonance in Med*. 2025;94(5):1946-1962. doi:10.1002/mrm.30601

6.	Leitão D, Tomi‐Tricot R, Bridgen P, et al. Parallel transmit pulse design for saturation homogeneity ( PUSH ) for magnetization transfer imaging at 7T. *Magnetic Resonance in Med*. 2022;88(1):180-194. doi:10.1002/mrm.29199

7.	Drago JM, Guerin B, Stockmann JP, Wald LL. Multiphoton parallel transmission (MP‐pTx): Pulse design methods and numerical validation.
